# Nitric Oxide-mediated *S*-nitrosylation of the Energy Sensor KIN10 Regulates RNA Splicing and Gene Expression in *Arabidopsis*

**DOI:** 10.1016/j.mcpro.2025.101459

**Published:** 2025-11-25

**Authors:** Yanyan Yi, Xiahe Huang, Wan Wang, Yingchun Wang, Jianru Zuo, Hongyan Guo

**Affiliations:** 1 State Key Laboratory of Seed Innovation, Institute of Genetics and Developmental Biology, Chinese Academy of Sciences, Beijing, China; 2 College of Advanced Agricultural Sciences, University of Chinese Academy of Sciences, Beijing, China; 3 Institute of Genetics and Developmental Biology, Chinese Academy of Sciences, Beijing, China

**Keywords:** arabidopsis, energy sensor, gene expression, KIN10, nitric oxide, phosphorylation, *S*-nitrosylation, spliceosome

## Abstract

Nitric oxide (NO) is a crucial signaling molecule involved in various developmental processes and stress responses through post-translational protein modification and modulation of gene expression. Despite significant advances in understanding the mechanism of NO-mediated protein modifications, how NO regulates gene expression remains largely unclear. Here, we show that the energy sensor KIN10, a catalytic α-subunit of sucrose non-fermenting 1-related kinase 1, plays a vital role in NO-mediated regulation of gene expression in *Arabidopsis*. NO-mediated *S*-nitrosylation at Cys-177 of KIN10 inhibits its degradation, leading to protein stabilization. A non-nitrosylatable mutation of Cys-177 to serine results in NO insensitivity and functional deficiencies. Quantitative phosphoproteomic analysis reveals that *S*-nitrosylation at Cys-177 of KIN10 modulates the phosphorylation of splicing factors within the spliceosome. We propose that NO regulates RNA splicing through the enhancement of KIN10 activity via *S*-nitrosylation, thereby establishing a molecular link between NO signaling and gene expression.

## Highlights

• KIN10 acts as a positive regulator of nitric oxide signaling.
• *S*-nitrosylation at Cys-177 stabilizes KIN10 protein and enhances its activity.
• NO-mediated *S*-nitrosylation of KIN10 modulates the phosphorylation status of splicing factors within the spliceosome complex.
• KIN10 bridges NO signaling and gene expression regulation.

## Graphical Abstract

**Figure:**
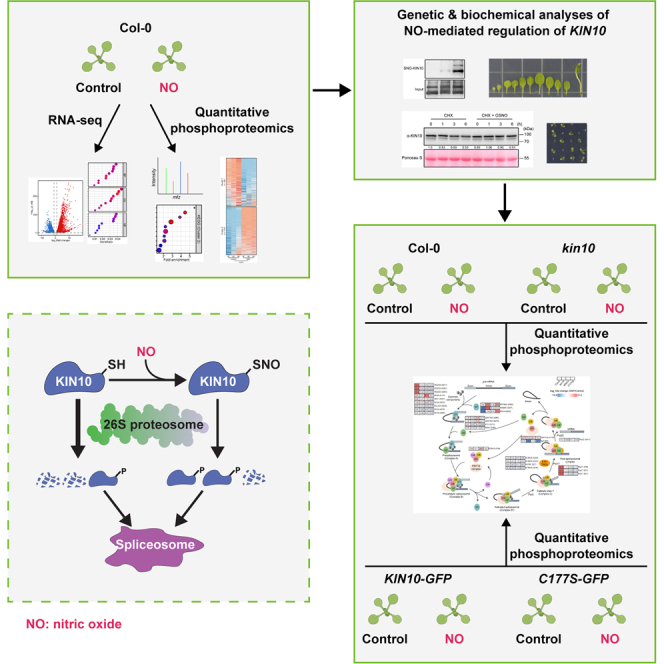

## In Brief

Nitric oxide (NO) is a key signaling molecule, but how it regulates gene expression is not well understood. Here we show that NO‑dependent *S*‑nitrosylation of the energy sensor KIN10 stabilizes the protein and alters the phosphorylation of splicing factors in Arabidopsis. *kin10* mutant is insensitive to NO and functionally impaired. These findings reveal a molecular link between NO signaling, KIN10 activity, and RNA splicing–mediated regulation of gene expression.

Nitric oxide (NO) is a critical signaling molecule that governs diverse physiological processes across both animal and plant kingdoms. In mammals, NO was initially identified as an endothelium-derived relaxing factor that induces relaxation in vascular smooth muscle tissues (1, 2). Subsequent studies have revealed its pleiotropic regulatory functions in numerous biological processes, including reproduction, neurotransmission, inflammation, apoptosis, and carcinogenesis (3). Similarly, in higher plants, NO serves as an essential signaling molecule that coordinates various physiological and pathological events, such as seed germination, root development, stomatal movement, flowering, fruit ripening, and responses to environmental stresses (4, 5, 6).

The primary mechanism through which NO executes its physiological effects is via post-translational modifications of proteins, including *S*-nitrosylation, tyrosine nitration, and metal nitrosylation (3, 4). Among these, *S*-nitrosylation is recognized as the predominant signaling pathway for NO in plants. It is a selective and reversible redox-based modification characterized by the covalent addition of an NO group to the reactive cysteine thiol of a protein, leading to the formation of an *S*-nitrosothiol (7). This process is mainly modulated by the intracellular levels of *S*-nitrosoglutathione (GSNO), a major bioactive NO species. Similar to other types of post-translational modifications, *S*-nitrosylation can alter protein structure, biochemical activity, subcellular localization, and protein-protein interactions (8). In higher plants, many *S*-nitrosylated proteins have been identified and are implicated in the regulation of various developmental processes and stress responses (9, 10, 11, 12, 13). Another important way through which NO exerts its biological functions is the modulation of gene expression, particularly under stress conditions (14, 15, 16, 17, 18, 19, 20). However, the precise molecular mechanisms underlying NO-mediated regulation of gene expression remain largely unclear.

Sucrose non-fermenting 1 (SNF1)-related kinase 1 (SnRK1) is a central metabolic regulator in plants, orchestrating the balance between energy-consuming anabolic pathways and energy-producing catabolic pathways (21, 22). SnRK1 belongs to an evolutionarily conserved family of serine/threonine protein kinases, which also includes SNF1 in yeast and AMP-activated protein kinase (AMPK) in mammals. These kinases function as cellular energy sensors activated under conditions of energy limitation (23, 24). Once activated, they phosphorylate numerous downstream targets, including key transcription factors and metabolic enzymes, thereby promoting catabolic reactions while suppressing anabolic processes to restore cellular energy homeostasis (25, 26). Typically, AMPK/SNF1/SnRK1 kinases operate as heterotrimeric complexes composed of one α-catalytic subunit and two regulatory subunits, β and γ (27). In *Arabidopsis*, three genes encode the α-catalytic subunit isoforms of SnRK1. Among these, *KIN10* (*SnRK1α1*/*SnRK1.1*, *AT3G01090*) is broadly expressed and contributes significantly to the overall SnRK1 kinase activity, whereas *KIN11* (*SnRK1α2*/*SnRK1.2*, *AT3G29160*) exhibits a more spatially restricted expression pattern (28, 29). *KIN12* (*SnRK1α3*/*SnRK1.3*, *AT5G39440*) is considered a non-expressed pseudogene (29). Single knockout mutants of *kin10* or *kin11* remain viable, but the double mutant is lethal, highlighting their essential and overlapping functions (26, 30). KIN10 and KIN11 have been identified as central regulators of the transcriptome in stress and energy signaling (21, 26).

Activation of SnRK1 kinase requires phosphorylation at a conserved threonine residue within the activation T-loop of the catalytic subunit (Thr-175 in KIN10), mediated by upstream kinases known as SnRK1-activating kinases (SnAK1 and SnAK2), also termed geminivirus Rep-interacting kinases (GRIK1 and GRIK2) (26, 31, 32, 33). Trehalose 6-phosphate (T6P), a proxy for the cellular sugar status, directly binds to KIN10, weakening its affinity for SnAKs (34, 35, 36). Additionally, SUMOylation and ubiquitination target SnRK1 for proteasomal degradation (33, 37, 38, 39). Two clade A type 2C protein phosphatases (ABI1 and PP2CA), known repressors of the abscisic acid (ABA) signaling pathway, interact with SnRK1 α-catalytic subunit, inducing its dephosphorylation and subsequent inactivation (40, 41). The kinase activity of KIN10 is also sensitive to redox status *in vitro* (42). These findings underscore the critical role of post-translational modifications in regulating KIN10 activity.

In this study, we demonstrate that *S*-nitrosylation of KIN10 enhances its protein stability and kinase activity, thereby transmitting NO signals to downstream targets, including splicing factors in the spliceosome. These findings uncover a novel mechanism by which NO and the energy sensor KIN10 synergistically regulate downstream gene expression.

## Experimental Procedures

### Plant Materials and Growth Conditions

The Arabidopsis (*Arabidopsis thaliana*) wild-type accession Columbia-0 (Col-0) and related mutants were used in this study. Seeds of *kin10*, *kin11* (WiscDsLox384F5), and *nox1* mutant were kindly provided by Yan Xiong, Ming-Yi Bai, and Yikun He (43, 44), respectively. Transgenic Arabidopsis plants were generated using *Agrobacterium*-mediated transformation as previously described (45).

Seeds were surface-sterilized, sown on 1/2 MS medium agar plates supplied with or without sucrose as indicated and imbibed at 4 °C for 2 days. The plates were then placed at 21 °C under a cycle of 16 h light and 8 h dark.

### Plasmid Construction

The pMAL2CGW-KIN10 vector was provided by Ming-Yi Bai (44). Site-directed mutagenesis was performed using specific PCR primer pairs to generate pMAL2CGW-KIN10^C133S^ and pMAL2CGW-KIN10^C177S^ constructs.

A genomic fragment containing both the putative promoter region (2047 bp) and coding sequence (2704 bp) of *KIN10* was amplified from the genomic DNA of wild-type Col-0 and cloned into the pGEM-T Easy vector (Promega), generating the construct pGEM-T-*proKIN10*::*KIN10*. Site-directed mutagenesis using specific PCR primer pairs was carried out to generate pGEM-T-*proKIN10*::*KIN10*^*C133S*^, pGEM-T-*proKIN10*::*KIN10*^*C177S*^, and pGEM-T-*proKIN10*::*KIN10*^*T175A*^ constructs. The resulting *proKIN10*::*KIN10* fragment and its mutated forms were subsequently cloned into the *Sal*I/*Xma*I sites of pCAMBIA1300-GFP-NosT and pCAMBIA1300-FLAG-NosT binary vectors, respectively.

All constructs were confirmed by DNA sequencing. The PCR primers used in this study are listed in Supplemental Table S10.

### Chemical Treatment

For sodium nitroprusside (SNP) treatment, seeds from the same batch were germinated and grown on sucrose-free 1/2 MS agar plates containing various concentrations of SNP as indicated. For sucrose treatment assays, seeds from the same batch were germinated and grown on 1/2 MS agar plates containing different concentrations of sucrose. Cotyledon greening rates were scored and analyzed accordingly.

NO vapor treatment assays were performed as previously described (46) with minor modifications. Briefly, 10 ml of 1% agarose with or without 2 mM SNP was placed on the lid of Petri dishes to release NO vapor for treatment.

### Purification of Recombinant Proteins

The pMAL2CGW-KIN10, pMAL2CGW-KIN10^C133S^, and pMAL2CGW-KIN10^C177S^ expression vectors were transformed into *Escherichia coli* strain BL21 (DE3). Expression and purification of the recombinant proteins were performed according to the manufacturer’s instructions (New England Biolabs).

### *In vitro S*-nitrosylation Assay

*In vitro S*-nitrosylation assay was performed as described (11) with minor modifications. Briefly, purified recombinant proteins were treated with the NO donor GSNO to induce *S*-nitrosylation, while free thiols in unmodified cysteines were blocked by an alkylating reagent. The nitrosothiol bonds were then selectively decomposed by ascorbate, allowing biotin labeling of the newly formed thiols for subsequent detection with an anti-biotin antibody. Approximately 30 μg of purified MBP-KIN10 recombinant proteins were incubated with either 200 μM GSNO (Sigma-Aldrich, Cat # N4148) or GSH at 23 °C for 40 min in the dark. After incubation, proteins were precipitated using cold acetone, washed three times with 70% acetone, and subsequently resuspended in blocking buffer [250 mM HEPES (pH 7.7), 4 mM EDTA, 0.1 mM neocuproine, 2.5% SDS, and 200 mM *N*-ethylmaleimide]. The sample was incubated at 50 °C for 40 min with frequent vortexing, followed by precipitation again with cold acetone and washing with 70% acetone. The resulting protein pellets were resuspended in HENS buffer [250 mM HEPES (pH 7.7), 4 mM EDTA, 0.1 mM neocuproine, and 1% SDS], and incubated with 5 mM sodium ascorbate and 0.33 mM biotin-maleimide for 1 h at room temperature. All reactions were carried out in the dark. Finally, aliquots of the sample were analyzed by SDS-PAGE without boiling, followed by either direct in-gel tryptic digestion for mass spectrometry analysis using a Thermo Fisher Scientific LTQ Orbitrap Elite mass spectrometer (47) or transfer to membranes for immunoblot analysis using an anti-biotin antibody (Cell Signaling Technology, Cat # 7075).

### *In vivo S*-nitrosylation Assay

*In vivo S*-nitrosylation assay was performed essentially as described previously (48). Briefly, plant samples were ground in liquid nitrogen and immediately extracted with HEN2 buffer [250 mM HEPES (pH 7.7), 1 mM EDTA, 0.1 mM neocuproine, 0.5% NP-40, 1 mM PMSF, and protease inhibitor cocktail]. The extracts were centrifuged at 13,000 rpm for 20 min at 4 °C, and the supernatant was collected. Approximately 200 μg of protein extracts were incubated with blocking buffer (see above) at 50 °C for 40 min. Subsequently, proteins were precipitated with cold acetone, washed three times with 70% acetone, and resuspended in 240 μl of HEN buffer supplemented with 1% SDS. Labeling was performed by adding 30 μl of 50 mM sodium ascorbate and 30 μl of 4 mM biotin-maleimide, followed by incubation for 1 h at room temperature. Proteins were then precipitated again and washed with 70% acetone. The resulting pellet was dissolved in 300 μl of HENS buffer and neutralized by adding 810 μl of neutralization buffer [25 mM HEPES (pH 7.7), 100 mM NaCl, 1 mM EDTA, and 0.5% Triton X-100]. Subsequently, 30 μl of streptavidin beads (Thermo Fisher Scientific, Cat # 29202) were added, and the mixture was incubated overnight at 4 °C. All procedures described above were conducted in the dark. The beads were washed six times with washing buffer [25 mM HEPES (pH 7.7), 600 mM NaCl, 1 mM EDTA, and 0.5% Triton X-100]. Finally, proteins were eluted and analyzed by immunoblotting.

### DAN Assay

The number of *S*-nitrosylated cysteine residues was measured using the 2,3-diaminonaphthalene (DAN) assay, essentially as described previously (49). Briefly, 200 μg of MBP-KIN10 recombinant protein was incubated with 200 μM GSNO at room temperature for 1 h in the dark. Free GSNO was subsequently removed using Zeba Spin Desalting Columns (Thermo Fisher Scientific, Cat # 89883) pre-equilibrated with PBS. The purified sample was then mixed with 300 μl of a reaction solution containing 200 μM HgCl_2_ and 200 μM DAN, followed by incubation for 30 min to convert DAN into fluorescent 2,3-naphthyltriazole (NAT). The reaction was terminated by adding 15 μl of 2.8 M NaOH. All the above steps were conducted in the dark. Fluorescence emitted by NAT was measured using a NanoDrop 3300 fluorospectrometer (Thermo Fisher Scientific), with an excitation wavelength of 365 ± 10 nm and an emission wavelength of 450 nm.

### Phosphopeptide Enrichment and Mass Spectrometry

The ground powder was lysed in protein extraction buffer [4% sodium deoxycholate (Sigma-Aldrich, Cat # D6750), 100 mM Tris-HCl (pH 8.5)]. The lysate was incubated at 100 °C for 10 min, followed by centrifugation at 12,000 × *g* for 10 min at room temperature to remove insoluble debris. Protein concentrations were measured using a BCA protein assay kit (Thermo Fisher Scientific, Cat # A55860). Subsequently, proteins were reduced and alkylated using 10 mM Tris (2-carboxyethyl) phosphine hydrochloride (Aladdin, Cat # T107252) and 40 mM 2-chloroacetamide (Sigma-Aldrich, Cat # 22790) at 45 °C for 5 min. Proteins were then digested overnight at 37 °C with sequencing-grade trypsin (Enzyme & Spectrum, Cat # P01001). After digestion, phosphopeptides were enriched using the GreenPhos method (50).

For LC-MS/MS analyses, the resuspended peptides were analyzed using an Orbitrap Fusion Lumos Tribrid mass spectrometer (Thermo Fisher Scientific) coupled online to an Easy-nLC 1000 (Thermo Fisher Scientific) in data-dependent acquisition mode. Peptides were separated by reverse-phase LC using an analytical column [150 μm (ID) × 250 mm (length)] packed with C18 particles of 1.9 μm diameter. The mobile phases for the LC consisted of buffer A (0.1% formic acid in H_2_O) and buffer B (0.1% formic acid in acetonitrile). A 110-min nonlinear gradient was used for the separation. All MS measurements were conducted in positive ion mode. Precursor ions were measured in the Orbitrap analyzer at a resolution of 240,000 (at 200 *m*/*z*) and a target value of 1 × 10^6^ ions. The 20 most intense ions from each MS scan were isolated, fragmented by high-energy collisional dissociation, and detected in the linear ion trap.

### Mass Spectrometry Data Analysis

The database search was performed on all raw MS files using MaxQuant software (version 2.6.1.0) (https://www.maxquant.org/maxquant). The search was conducted against the *A. thaliana* protein sequence database downloaded from UniProt (accessed 05-Nov-2023, containing 39,284 entries). The database search parameters were set up as follows: the digestion mode was trypsin/P; the maximum missed cleavages was 2; the mass tolerance for precursor ions was 4.5 ppm; the mass tolerance for fragment ions was 0.5 Da; the minimum score for unmodified peptides was 15; the minimum score for modified peptides was 40; variable modifications included phosphorylation on STY, N-terminal acetylation, and methionine oxidation; the fixed modification included cysteine carbamidomethylation. The false discovery rates for both peptide and protein identifications were set to 0.01. All other parameters were set to default values.

Bioinformatics and statistical analyses were primarily performed using Perseus (version 2.0.11) (51, 52). For analysis of differential phosphosites levels, statistical significance was determined using a two-tailed *t*-test with *p* < 0.05, and biological significance was defined by a fold change threshold of 1.5. Pathway enrichment analysis was conducted using the Kyoto Encyclopedia of Genes and Genomes (KEGG) pathway database (version 99.1) (https://www.kegg.jp/kegg/pathway.html) (53). To identify sequence motifs associated with different types of phosphorylation, MOMO (version 5.5.8) (https://meme-suite.org/meme/tools/momo) was employed to analyze motifs in phosphopeptides that were upregulated following NO treatment (54). Functional annotation of identified proteins was performed through Gene Ontology (GO) enrichment analysis using the Database for Annotation, Visualization, and Integrated Discovery (DAVID, version 6.8). Data visualization, including volcano plots and hierarchical clustering analyses, was performed using PTMCloud tools (https://www.ptm-biolab-css.com.cn/cloud/cloudTool).

### Experimental Design and Statistical Rationale

For label-free quantitative phosphoproteomic analysis, equal amounts of proteins from seedlings treated with or without 2 mM SNP applied on the lid of the Petri dishes for 5 h were separately subjected to enzymatic digestion and phosphopeptide enrichment, followed by LC-MS/MS analysis. Each treatment was analyzed with three independent biological replicates. The phosphopeptides were identified and quantified using MaxQuant software (version 2.6.1.0). Phosphosites were retained for statistical analysis only if they were quantified with two valid intensity in at least one treatment group (55). Fuzzy C-Means clustering analysis was performed to identify phosphosites with differential abundance levels between treatments.

For other quantification analyses, data are presented as mean ± SD and compared to the respective control groups. Statistical significance (*p*-value unless otherwise specified) was determined using one-way ANOVA with Tukey’s HSD post hoc test or two-tailed Student’s *t*-test as indicated in the figure legends. ∗*p* < 0.05, ∗∗*p* < 0.01, ∗∗∗*p* < 0.001. All experiments were repeated at least 3 times, and representative results are presented.

## Results

### *KIN10* Positively Regulates NO Signaling

**Fig. 1:**
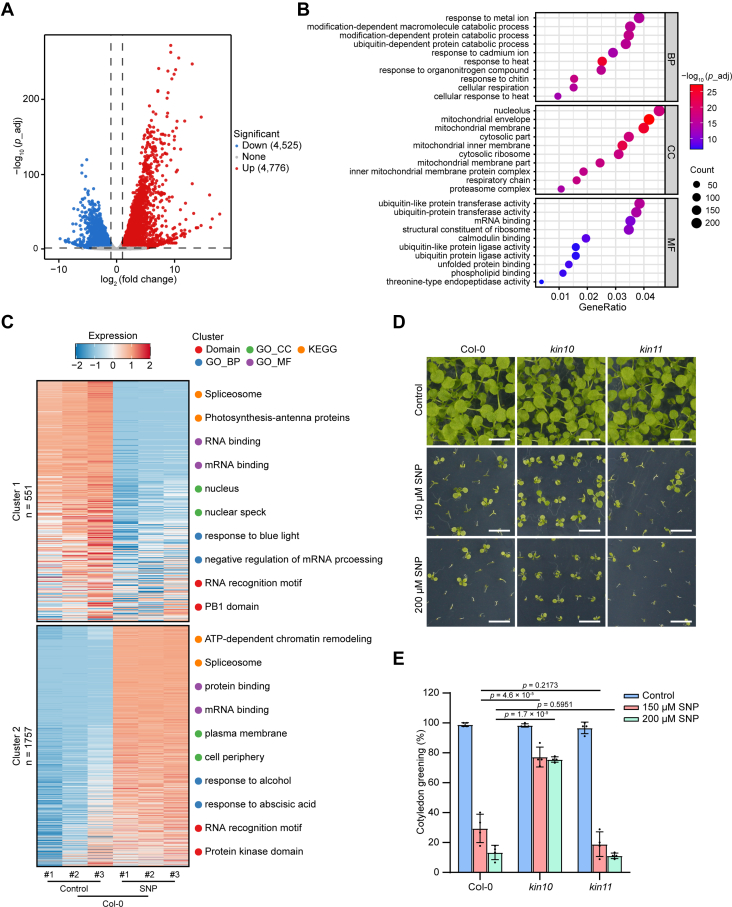
***KIN10* positively regulates NO signaling.***A*, volcano plot of differentially expressed genes (DEGs) between sodium nitroprusside (SNP) treatment and control conditions. Twelve-day-old wild-type (Col-0) seedlings were treated with or without (control) 2 mM SNP applied on the lid of the Petri dishes for 5 h. *Red* (upregulated) and *blue* (downregulated) dots represent DEGs with expression changes exceeding 2.0-fold (adjusted *p* < 0.05). *B*, top 10 significantly enriched Gene Ontology (GO) terms for biological process (BP), cellular component (CC), and molecular function (MF) categories derived from the upregulated DEGs shown in (*A*). *C*, clustered heatmap of phosphosites showing significant differences between SNP treatment and control, based on log_2_ of *z*-score intensities (*p* < 0.05). Twelve-day-old Col-0 seedlings were treated with or without (control) 2 mM SNP applied on the lid of the Petri dishes for 5 h. The top 2 most significantly enriched terms from GO_BP, GO_CC, GO_MF, KEGG pathway, and protein domain enrichment analyses are presented on the *right*. *D*, sixteen-day-old wild-type (Col-0), *kin10*, and *kin11* mutant seedlings germinated and grown on 1/2 MS agar plates with or without (control) the indicated concentrations of SNP. Scale bars, 0.5 cm. *E*, quantitative analysis of the cotyledon greening rate of seedlings shown in (*D*) (n = 180). Data are presented as mean ± SD, one-way ANOVA with Tukey’s HSD test.

Nitric oxide (NO) is an important regulator of growth and stress responses in plants, functioning through post-translational modifications of target proteins and influencing gene expression (4, 17). To investigate how NO affects gene expression, we compared the transcriptomes from seedlings of wild-type *Arabidopsis* Columbia-0 (Col-0) subjected to either treatment with the NO donor SNP or control conditions. RNA-seq analysis identified a total of 9301 differentially expressed genes (DEGs) (Fig. 1*A* and Supplemental Table S1). GO analysis of these DEGs indicated the involvement of NO in diverse biological processes, including responses to stress, catabolic, and biosynthetic processes (Fig. 1*B*, Supplemental Fig. S1*A*, and Supplemental Table S2), highlighting the pivotal roles of NO in mediating energy homeostasis and stimulus responses.

In living cells, protein phosphorylation plays a crucial role in signal transduction and gene expression. To investigate the relationship between NO signaling and protein phosphorylation, we performed a quantitative phosphoproteomic analysis of Col-0 seedlings treated with SNP or control conditions using LC-MS/MS. This analysis identified a total of 11,804 unique phosphosites from 3233 phosphoproteins, among which 3232 phosphosites exhibited significant differences between SNP treatment and the control (*p* < 0.05, fold change >1.5 or fold change <0.67) (Supplemental Fig. S1, *B*–*D* and Supplemental Table S3), suggesting that NO signaling induces global phosphorylation alterations. Using hierarchical clustering analysis, the identified phosphosites can be categorized into 2 clusters based on their *z*-score intensities, corresponding to NO-downregulated and NO-upregulated phosphosites, respectively (Fig. 1*C* and Supplemental Table S4). GO terms and KEGG pathways enrichment analyses of the phosphoproteins in both clusters revealed significant enrichment in spliceosome-related and mRNA processing processes (Fig. 1*C*, Supplemental Fig. S1, *E* and *F*, and Supplemental Table S4), reminiscent of AMPK/SnRK1’s role in regulating RNA splicing (56, 57, 58, 59, 60). To extract phosphorylation motifs from the dataset, the 2505 phosphosites that were at least 50% more abundant upon NO treatment were submitted to Motif-X (https://meme-suite.org/meme/tools/momo) (54). This analysis identified 22 conserved motifs, including 19 phosphoserine (p-Ser) motifs and 3 phosphothreonine (p-Thr) motifs (Table 1), 4 of which (motifs 4, 6, 8, and 9) matched the characteristics of the SnRK1 substrate motifs (30) and accounted for 43.7% of the NO upregulated phosphopeptides population, suggesting that SnRK1 is important in NO signaling.

**Table 1:** Phosphorylation motifs significantly enriched in NO-upregulated phosphopeptides identified by Motif-X

Motif No.	Motif	Motif	Foreground Matches	Fold	*p* value
		Score		Increase	
1	xxxxxxx_S_PRxxxxx	288.62	78	12.5	1.50E-56
2	xxxxxxx_S_PKxxxxx	242.3	68	12.1	1.60E-48
3	xxxxxxx_S_PxRxxxx	199.56	62	10.4	1.20E-40
4	xxxxxxx_S_Pxxxxxx	165.14	624	5.6	6.10E-278
5	xxxxRSx_S_xxxxxxx	64.93	99	7	1.50E-49
6	xxxxxxx_S_DxExxxx	64.51	72	7.8	2.90E-39
7	xxxxxxG_S_xxxxxxx	30.71	251	1.9	4.10E-21
8	xxxxxxx_S_DxDxxxx	39.57	48	5.9	1.40E-21
9	xxxxRxx_S_xxxxxxx	22.96	287	2.5	1.40E-44
10	xxxxxxx_S_ExExxxx	28.96	47	4.1	1.90E-15
11	xxDxxxD_S_xxxxxxx	23.21	28	4.5	1.30E-10
12	xxxxxxx_S_ExDxxxx	21.4	31	4.2	8.30E-11
13	xxxxxxx_S_xxGxxxx	10.9	216	1.6	5.70E-12
14	xxxxxxx_S_xPxxPxx	17.57	41	4.3	2.80E-14
15	xxxxSxx_S_xxxxxxx	9.48	308	1.3	3.80E-06
16	xxxxxSx_S_xxxxxxx	10.28	386	1.5	8.30E-17
17	xxxxxDx_S_xxxxxxx	7.15	163	1.4	2.90E-06
18	xxxxxNx_S_xxxxxxx	6.31	131	1.4	6.00E-05
19	xxxxxxx_S_xDxxxxx	6.61	188	1.7	1.80E-12
20	xxxxxPx_T_Pxxxxxx	66.43	29	28.3	2.00E-32
21	xxxxxxx_T_Pxxxxxx	34.72	94	7.8	7.00E-58
22	xxxxxxx_T_Exxxxxx	8.04	30	2.1	1.60E-04

Motifs 1 to 19 represent phosphoserine motifs, while motifs 20 to 22 corresponds to phosphothreonine motifs. The motif width was set to 15, with a minimum occurrence threshold of 20 for each residue/position pair. Foreground matches represent the number of foreground peptides that match the motif.

Given that *S*-nitrosylation is an important mechanism through which NO executes its physiological functions and that global phosphorylation changes occur in NO signaling, we next investigated whether the *S*-nitrosylation of kinase protein(s) contributes to the regulation of gene expression in NO signaling. In a site-specific nitrosoproteomics study, we focused on kinase proteins associated with the energy and stimulus responses and identified a peptide that was mapped to KIN10 and KIN11 (Supplemental Fig. S2), the catalytic α-subunits of SnRK1 (61). This finding is consistent with the motif enrichment of the quantitative phosphoproteomic analysis above (Table 1). Previous studies have demonstrated that high concentrations of SNP inhibit both shoot and root growth in plants (43, 62). To analyze the regulatory role of NO in plant growth and development, we sowed seeds on medium supplemented with SNP and observed that SNP treatment at concentrations above 100 μM significantly suppressed the growth and development of wild-type Col-0 (Supplemental Fig. S1*G*). To explore whether *KIN10* or *KIN11* is involved in NO signaling, we analyzed the phenotype of *kin10* and *kin11*, two null alleles of *KIN10* and *KIN11*, under SNP treatment. In a cotyledon greening assay, we observed that 150 μM and 200 μM SNP severely inhibited the development of both Col-0 and *kin11* seedlings, but the growth-repressive effects were markedly alleviated in *kin10* (Fig. 1, *D* and *E*). Collectively, these results suggest that *KIN10* is a critical positive regulator of NO signaling.

### KIN10 Undergoes *S*-nitrosylation *in vitro* and *in vivo*

**Fig. 2:**
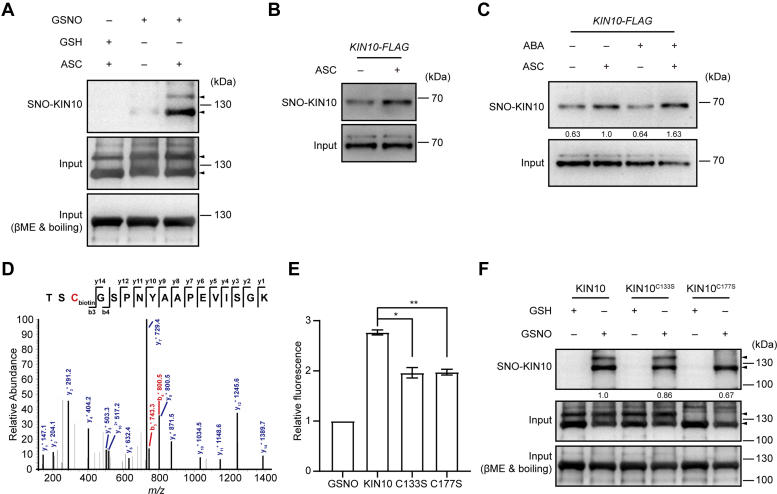
**KIN10 is *S*-nitrosylated.***A*, biotin-switch analysis of *S*-nitrosylated MBP-KIN10 recombinant protein (SNO-KIN10) *in vitro* in the presence or absence of the reductant sodium ascorbate (ASC). Sample treated with reduced GSH served as a negative control. *S*-nitrosylated proteins were detected with an anti-biotin antibody under non-reducing conditions (*top panel*). Input samples under non-reducing conditions and reducing conditions (with β-mercaptoethanol (βME) and boiling) are shown in the *middle* and *bottompanels*, respectively. *B*, analysis of *S*-nitrosylated KIN10 protein (SNO-KIN10) in 7-day-old *KIN10-FLAG*/*kin10* transgenic seedlings using the biotin-switch method. Sample without ASC treatment served as a negative control. Both total (*bottom panel*) and *S*-nitrosylated (*top panel*) KIN10 were detected using anti-KIN10 antibody. *C*, analysis of *S*-nitrosylated KIN10 protein (SNO-KIN10) in 7-day-old *KIN10-FLAG*/*kin10* transgenic seedlings treated with or without 50 μM ABA for 30 min using the biotin-switch method. Samples without ASC treatment served as negative controls. Both total (*bottom panel*) and *S*-nitrosylated (*top panel*) KIN10 were detected using anti-KIN10 antibody. Quantification is shown below the blot. The relative level of *S*-nitrosylated KIN10 in *KIN10-FLAG* transgenic seedlings without ABA treatment (with ASC) is set as 1.0. *D*, mass spectrometric analysis of tryptic fragments from GSNO-treated MBP-KIN10 recombinant protein. The b- and y-type product ions are indicated, with Cys-177 identified as an *S*-nitrosylated residue charged with biotin. *E*, DAN analysis of *S*-nitrosylated residues in the GSNO tripeptide, MBP-KIN10, MBP-KIN10^C133S^, and MBP-KIN10^C177S^ recombinant proteins. The relative *S*-nitrosylation level of GSNO is set as 1.0. Data are presented as mean ± SD, two-tailed Student’s *t*-test, ∗∗*p* < 0.01, ∗*p* < 0.05. *F*, analysis of *S*-nitrosylated MBP-KIN10, MBP-KIN10^C133S^, and MBP-KIN10^C177S^ recombinant proteins using the *in vitro* biotin-switch method. *S*-nitrosylated proteins were detected with an anti-biotin antibody under non-reducing conditions (*top panel*). Input samples under non-reducing conditions and reducing conditions (with βME and boiling) are shown in the *middle* and *bottom panels*, respectively. Quantification is shown below the blot. The relative level of *S*-nitrosylated MBP-KIN10 treated with GSNO is set as 1.0.

KIN10 was previously identified as a putative *S*-nitrosylated protein in a nitrosoproteomic study (61). We therefore tested whether KIN10 undergoes *S*-nitrosylation using a biotin-switch assay. This method involves replacing NO groups attached to cysteine residues in *S*-nitrosylated proteins with biotin molecules, which can then be detected using biotin-specific antibodies. The *in vitro* biotin-switch assay demonstrated that the MBP-KIN10 recombinant protein was *S*-nitrosylated by GSNO but not by reduced GSH, which served as a negative control (Fig. 2*A*). To analyze the *S*-nitrosylation of KIN10 in planta, we generated transgenic plants carrying the *pKIN10*::*KIN10-FLAG* transgene in the *kin10* mutant background and observed *S*-nitrosylation of KIN10 under normal growth conditions (Fig. 2*B*). Given that ABA is a key phytohormone controlling stress responses and rapidly triggers NO bursts (63, 64), we analyzed the *S*-nitrosylation of KIN10 following ABA treatment and found it to be significantly enhanced (Fig. 2*C*). KIN10 is highly conserved across various kingdoms and contains 8 cysteine (Cys, C) residues. Among these, Cys-154 and Cys-177 were predicted to be *S*-nitrosylated using GPS-SNO software (http://sno.biocuckoo.org/) (65). To identify the *S*-nitrosylated residues of KIN10, the tryptic peptides derived from the MBP-KIN10 recombinant protein treated with GSNO were subjected to LC-MS/MS. This analysis identified Cys-133, Cys-177, and Cys-418 as *S*-nitrosylated residues (Fig. 2*D*, Supplemental Figs. S2, and S3). Since Cys-154 and Cys-418 are not conserved across species (Supplemental Fig. S2), we focused our subsequent analysis on the conserved cysteines Cys-133 and Cys-177.

In addition, to quantify the number of *S*-nitrosylated Cys residues in KIN10, we performed an assay that measures the conversion ratio of 2,3-diaminonaphthalene (DAN) into fluorescent 2,3-naphthyltriazole (NAT) catalyzed by NO released from thiol groups (66). The MBP-KIN10 recombinant protein exhibited an approximately 3-fold higher DAN-NAT conversion ratio compared to the GSNO tripeptide, which contains a single *S*-nitrosylated Cys residue (Fig. 2*E*). Notably, substitution of Cys-133 or Cys-177 with a serine (Ser, S) residue (KIN10^C133S^ or KIN10^C177S^) resulted in a significantly reduced conversion ratio (Fig. 2*E*), indicating that at least 3 Cys residues of KIN10 can be *S*-nitrosylated. Consistently, *S*-nitrosylation of both MBP-KIN10^C133S^ and MBP-KIN10^C177S^ was substantially reduced compared to that of MBP-KIN10 recombinant protein (Fig. 2*F*). We noticed that two bands were detected in both the input and *S*-nitrosylated KIN10 proteins under non-reducing conditions (Fig. 2, *A* and *F*), and the C177S mutation eliminated the upper band, suggesting that Cys-177 is essential for the oxidation-dependent aggregation of KIN10. Taken together, these results demonstrate that KIN10 can be *S*-nitrosylated.

### NO positively regulates KIN10 stability

**Fig. 3:**
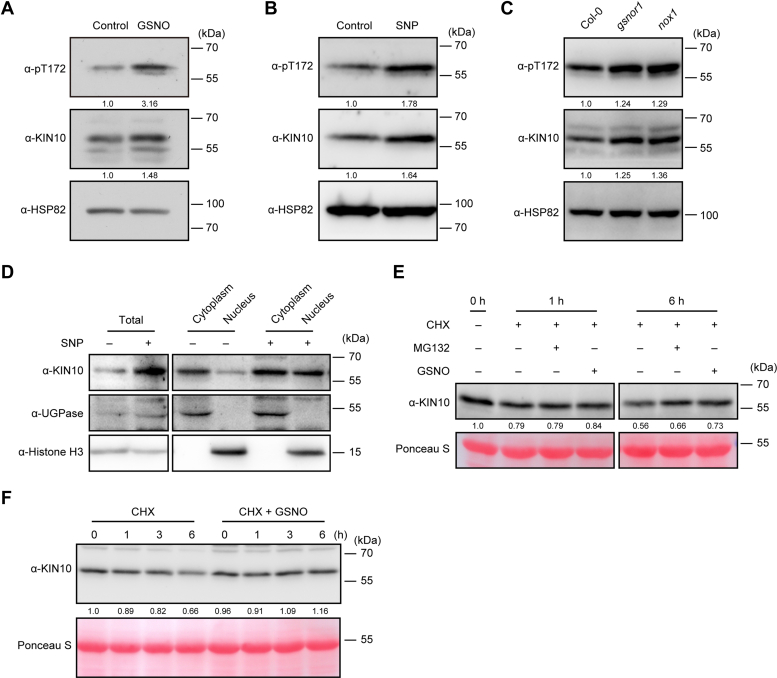
**NO positively regulates KIN10 stability.***A*, analysis of KIN10 accumulation and phosphorylation in 12-day-old wild-type (Col-0) seedlings treated with or without (control) 200 μM GSNO for 6.5 h. Quantification is shown below the blot. The relative levels of phosphorylated KIN10 and total KIN10 protein normalized to HSP82 in the absence of GSNO treatment are set as 1.0, respectively. *B*, analysis of KIN10 accumulation and phosphorylation in 12-day-old wild-type (Col-0) seedlings treated with or without (control) 2 mM SNP applied on the lid of the Petri dishes for 5 h. Quantification is shown below the blot. The relative levels of phosphorylated KIN10 and total KIN10 protein normalized to HSP82 in the absence of SNP treatment are set as 1.0, respectively. *C*, analysis of KIN10 accumulation and phosphorylation in 12-day-old wild-type (Col-0), *gsnor1*, and *nox1* mutant seedlings. Quantification is shown below the blot. The relative levels of phosphorylated KIN10 and total KIN10 protein normalized to HSP82 in Col-0 seedlings are set as 1.0, respectively. *D*, analysis of KIN10 protein in total, cytoplasmic, and nuclear fractions of 10-day-old Col-0 seedlings treated with or without 2 mM SNP applied on the lid of the Petri dishes for 12 h. *E*, cell-free protein degradation assay of KIN10 protein in extracts from Col-0 seedlings. Protein extracts from 10-day-old Col-0 seedlings were incubated with 100 μM cycloheximide (CHX) in the presence or absence of the proteasome inhibitor MG132 (50 μM) or GSNO (200 μM). Samples were collected at the indicated time points. Quantification is shown below the blot. The relative level of KIN10 normalized to the Ponceau S-stained Rubisco large subunit in the untreated sample is set as 1.0. *F*, cell-free protein degradation assay of KIN10 protein in extracts from Col-0 seedlings. Protein extracts from 10-day-old Col-0 seedlings were incubated with 100 μM CHX in the presence or absence of 200 μM GSNO. Samples were collected at the indicated time points. Quantification is shown below the blot. The relative level of KIN10 normalized to the Ponceau S-stained Rubisco large subunit in the untreated sample is set as 1.0. The phosphorylation and protein levels of KIN10 were assessed using anti-pT172 and anti-KIN10 antibodies, respectively. HSP82 and Ponceau S staining served as loading controls in (*A*–*C*, *E*, and *F*). Histone H3 and UGPase were used as nuclear and cytoplasmic markers, respectively, in (*D*).

Similar to other post-translational modifications, *S*-nitrosylation influences various properties of target proteins, including stability, subcellular localization, biochemical activity, and protein-protein interactions. We found that the transcription of *KIN10* was downregulated upon treatment with GSNO or *S*-nitrosocysteine (Cys-NO) (Supplemental Fig. S4*A*). However, the accumulation of KIN10 protein significantly increased following treatment with NO donors (Fig. 3, *A* and *B*, and Supplemental Fig. S4*B*). Phosphorylation of a conserved threonine (Thr, T) residue located in the T-loop of the catalytic domain is essential for the activity of AMPK/SNF1/SnRK1 kinase, specifically Thr-175 in KIN10 (31). Using polyclonal antibodies that recognize a human AMPKα phospho-T172 activation loop peptide (pT172 antibodies) (Supplemental Fig. S4*C*), we found that GSNO and SNP significantly enhanced the T-loop phosphorylation level of KIN10 (Fig. 3, *A*, and *B*). Consistently, both the accumulation of KIN10 and its T-loop phosphorylation were also substantially increased in *gsnor1* and *nox1*, two NO over-accumulating mutants (43, 67), compared to the WT (Fig. 3*C*, Supplemental Fig. S4, *D* and *E*). These results are consistent with a previous proteomic study showing that KIN10 protein levels are increased in the *gsnor1* mutant compared to the WT (68). Under normal growth conditions, KIN10 is localized in both cytoplasm and the nucleus, with stress triggering its translocation between these compartments (69, 70, 71). We found that NO treatment enhanced the accumulation of KIN10 in both cytoplasm and the nucleus (Fig. 3*D* and Supplemental Fig. S4*D*). Currently, we cannot exclude the possibility that NO directly regulates KIN10 kinase activity. Nevertheless, these results suggest that NO plays a role in regulating the stability of KIN10 protein.

Previous studies have shown that KIN10 undergoes degradation via the 26S proteasome pathway (33, 37, 38). Consistently, a cell-free protein degradation assay showed that endogenous KIN10 protein was gradually degraded in total protein extracts from wild-type (Col-0) plants in the presence of cycloheximide (CHX), a protein biosynthesis inhibitor (Fig. 3, *E* and *F*). Notably, this degradation was significantly inhibited by treatment with the proteasome inhibitor MG132 or GSNO (Fig. 3, *E* and *F*). Collectively, these results suggest that NO positively regulates the stability of KIN10 protein through inhibiting its degradation.

### *S*-nitrosylation of KIN10 at Cys-177 Positively Regulates Protein Stability and Function

**Fig. 4:**
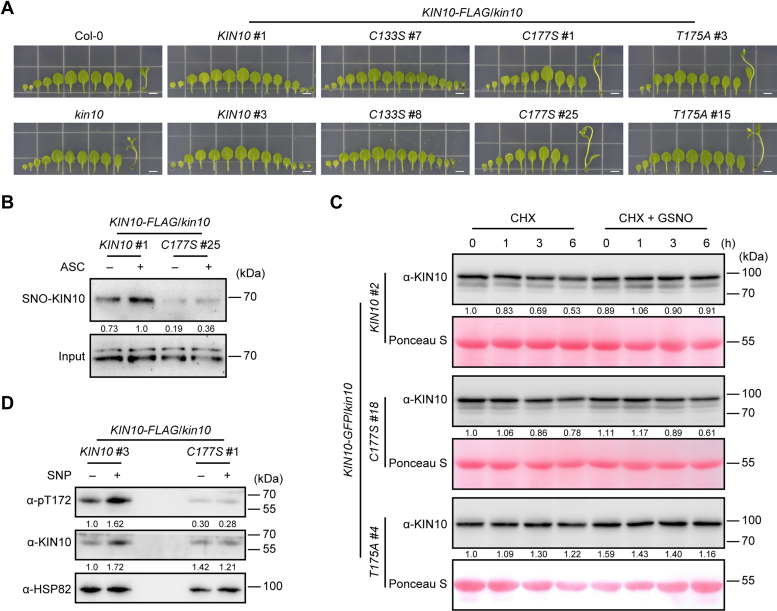
***S*-nitrosylation at Cys-177 positively regulates protein stability and activity of KIN10.***A*, rosette leaf series and the first bolt of 3-week-old wild-type (Col-0), *kin10*, and *kin10* seedlings carrying *pKIN10*::*KIN10-FLAG*, *pKIN10*::*KIN10*^*C133S*^*-FLAG*, *pKIN10*::*KIN10*^*C177S*^*-FLAG*, or *pKIN10*::*KIN10*^*T175A*^*-FLAG* transgenes, germinated and grown on 1/2 MS agar plates under long-day conditions (16 h light/8 h dark). Transgenic line numbers are indicated. Scale bars, 0.5 cm. *B*, analysis of *S*-nitrosylated KIN10-FLAG and KIN10^C177S^-FLAG in transgenic seedlings using the biotin-switch method. Quantification is shown below the blot. The relative level of *S*-nitrosylated KIN10 in *KIN10-FLAG* transgenic seedlings with ASC treatment is set as 1.0. *C*, cell-free protein degradation assay of KIN10 protein in extracts from *pKIN10*::*KIN10-GFP*, *pKIN10*::*KIN10^C177S^-GFP*, and *pKIN10*::*KIN10^T175A^-GFP* transgenic seedlings. Protein extracts from 10-day-old transgenic seedlings were incubated with 100 μM CHX in the presence or absence of 100 μM GSNO. Samples were collected at the indicated time points. Quantification is shown below the blot. The relative level of KIN10-GFP normalized to the Ponceau S-stained Rubisco large subunit in the untreated sample is set as 1.0. *D*, analysis of KIN10 accumulation and phosphorylation in 12-day-old *pKIN10*::*KIN10-FLAG* and *pKIN10*::*KIN10*^*C177S*^*-FLAG* transgenic seedlings treated with or without 2 mM SNP applied on the lid of the Petri dishes for 5 h. Quantification is shown below the blot. The relative levels of phosphorylated KIN10 and total KIN10 protein normalized to HSP82 in *KIN10-FLAG* transgenic seedlings in the absence of SNP treatment are set as 1.0, respectively. The phosphorylation and protein levels of KIN10 were assessed using anti-pT172 and anti-KIN10 antibodies, respectively, in (*B*–*D*). Ponceau S staining and HSP82 served as loading controls in (*C* and *D*), respectively.

To analyze the role of *S*-nitrosylation, we generated transgenic plants in the *kin10* mutant background carrying wild-type *KIN10*, *KIN10*^*C133S*^, or *KIN10*^*C177S*^ genomic DNA fragments fused to *FLAG* or *GFP* tags under the control of the native *KIN10* promoter. We also generated inactive KIN10 mutant transgenic plants with a substitution of the conserved Thr-175 residue in the T-loop to alanine (Ala, A) (KIN10^T175A^). Transgenic lines with comparable transgene expression levels were selected for subsequent experiments (Supplemental Fig. S5, *A* and *B*). Previous studies have shown that *KIN10* overexpression delays flowering under long-day conditions (26, 72, 73) and decreases sensitivity to high concentrations of sucrose (26). We found that the flowering time of *pKIN10*::*KIN10-FLAG* and *pKIN10*::*KIN10*^*C133S*^*-FLAG* transgenic plants was significantly delayed, whereas *pKIN10*::*KIN10*^*C177S*^*-FLAG* and *pKIN10*::*KIN10*^*T175A*^*-FLAG* transgenic plants exhibited flowering times similar to those of Col-0 and *kin10* (Fig. 4*A*, Supplemental Fig. S5*C*, and Table 2). In addition, high concentrations of sucrose inhibited the growth of *KIN10-FLAG* and *KIN10*^*C133S*^*-FLAG* transgenic plants but not that of the *kin10* mutant, *KIN10*^*C177S*^*-FLAG*, or *KIN10*^*T175A*^*-FLAG* transgenic plants (Supplemental Fig. S5, *D* and *E*). These results indicate that Cys-177 plays a critical role in the function of KIN10.

**Table 2:** *S*-nitrosylation of KIN10 at Cys-177 regulates flowering time

Genotype of plants	Days to bolting	Rosette leaf number
Col-0	20.5 ± 1.2	8.6 ± 0.8
*kin10*	20.7 ± 1.2	8.0 ± 0.6
*KIN10-FLAG* #1	22.8 ± 1.5	11.0 ± 0.7
*KIN10-FLAG* #3	23.8 ± 1.6	10.7 ± 0.8
*KIN10*^*C133S*^*-FLAG* #7	23.8 ± 1.7	10.3 ± 0.8
*KIN10*^*C133S*^*-FLAG* #8	21.3 ± 1.1	9.7 ± 0.8
*KIN10*^*C177S*^*-FLAG* #1	18.5 ± 0.9	8.2 ± 0.7
*KIN10*^*C177S*^*-FLAG* #25	18.6 ± 1.1	7.9 ± 0.8
*KIN10*^*T175A*^*-FLAG* #3	18.7 ± 1.0	7.7 ± 0.7
*KIN10*^*T175A*^*-FLAG* #15	18.7 ± 0.9	7.6 ± 0.6

In each genotype, 59 plants were analyzed.

We next investigated the potential regulatory role of Cys-177 on KIN10. The *S*-nitrosylation level of KIN10^C177S^-FLAG was found to be lower than that of the wild-type KIN10-FLAG protein in planta (Fig. 4*B*), demonstrating that Cys-177 of KIN10 is *S*-nitrosylated *in vivo*. Given that NO increases KIN10 protein stability by inhibiting its degradation (Fig. 3, *E* and *F*), we then asked whether *S*-nitrosylation at Cys-177 of KIN10 plays a role in this process. In the cell-free degradation assay, both KIN10-FLAG and KIN10^C177S^-FLAG proteins were gradually degraded in total protein extracts from transgenic plants in the presence of CHX (Fig. 4*C* and Supplemental Fig. S5*F*), whereas the inactive KIN10^T175A^-FLAG protein remained stable (Fig. 4*C* and Supplemental Fig. S5*F*). Upon treatment with GSNO, the degradation of KIN10-FLAG was substantially inhibited in *KIN10-FLAG* transgenic plants but not in *KIN10*^*C177S*^*-FLAG* transgenic plants (Fig. 4*C* and Supplemental Fig. S5*F*). Moreover, both NO-enhanced KIN10 stability and NO-enhanced T-loop phosphorylation were nearly abolished by the KIN10^C177S^ mutation in planta (Fig. 4*D*). Taken together, these results suggest that *S*-nitrosylation at Cys-177 plays a critical role in regulating protein stability and function of KIN10.

### *S*-nitrosylation of KIN10 at Cys-177 Regulates NO Signaling

**Fig. 5:**
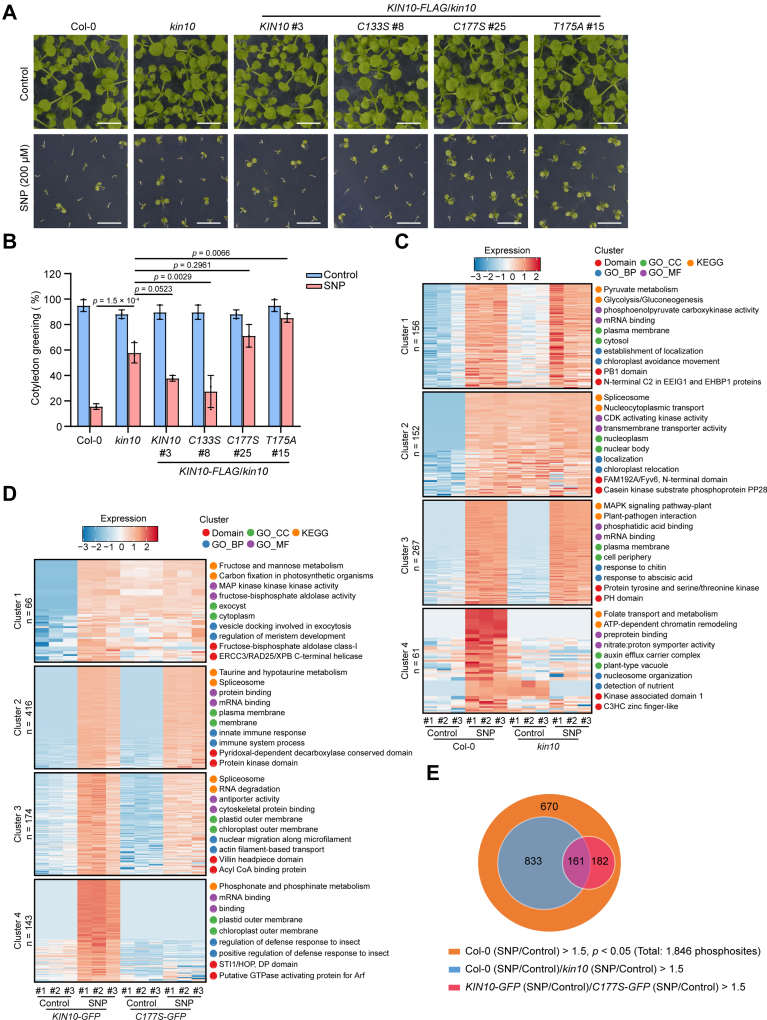
***S*-nitrosylation of KIN10 at Cys-177 regulates NO signaling.***A*, two-week-old seedlings of the indicated genotypes germinated and grown on 1/2 MS agar plates with or without (control) 200 μM SNP. Scale bars, 0.5 cm. *B*, quantitative analysis of the cotyledon greening rate of seedlings shown in (*A*) (n = 135). Data are presented as mean ± SD, one-way ANOVA with Tukey’s HSD test. *C*, clustered heatmap of phosphosites identified in both WT (Col-0) and *kin10* mutant, and significantly upregulated upon SNP treatment compared to control conditions in Col-0, based on log_2_ of *z*-score intensities (*p* < 0.05). Twelve-day-old Col-0 and *kin10* mutant seedlings were treated with or without (control) 2 mM SNP applied on the lid of the Petri dishes for 5 h. The top 2 most significantly enriched terms from GO_BP (biological process), GO_CC (cellular component), GO_MF (molecular function), KEGG pathway, and protein domain enrichment analyses are presented on the *right*. *D*, clustered heatmap of phosphosites identified in both *KIN10-GFP*/*kin10 kin11* and *KIN10^C177S^-GFP*/*kin10kin11* transgenic plants, and significantly upregulated upon SNP treatment compared to control conditions in *KIN10-GFP*/*kin10 kin11*, based on log_2_ of *z*-score intensities (*p* < 0.05). Twelve-day-old *KIN10-GFP* and *KIN10^C177S^-GFP* transgenic seedlings in *kin10 kin11* double mutant background were treated with or without (control) 2 mM SNP applied on the lid of the Petri dishes for 5 h. The most significantly enriched terms from GO_BP, GO_CC, GO_MF, KEGG pathway, and protein domain enrichment analyses are presented on the *right*. *E*, venn diagram of significantly upregulated phosphosites upon NO treatment in WT (Col-0), Col-0/*kin10*, and (*KIN10-GFP*/*kin10kin11*)/(*KIN10^C177S^-GFP*/*kin10kin11*) groups.

Data presented above suggest that *KIN10* is a critical regulator of NO signaling and that *S*-nitrosylation of Cys-177 positively regulates KIN10 stability. To assess the physiological role of *S*-nitrosylation at Cys-177 in NO signaling, we analyzed the phenotypes of wild-type Col-0, the *kin10* mutant, *KIN10-FLAG*, *KIN10*^*C133S*^*-FLAG*, *KIN10*^*C177S*^*-FLAG*, and *KIN10*^*T175A*^*-FLAG* transgenic plants in the *kin10* background in response to NO treatment. Upon treatment with high concentrations of SNP, the *kin10* mutant exhibited decreased sensitivity to SNP compared to the WT. This phenotype was partially rescued by the *KIN10-FLAG* and *KIN10*^*C133S*^*-FLAG* transgenes, but not by the *KIN10*^*C177S*^*-FLAG* or *KIN10*^*T175A*^*-FLAG* transgenes (Fig. 5, *A* and *B*), suggesting that *S*-nitrosylation of KIN10 at Cys-177 plays an important role in NO signal perception.

To further investigate the biological function of KIN10 and its *S*-nitrosylation at Cys-177 in NO signaling, we performed two independent quantitative phosphoproteomic analyses using LC-MS/MS for phosphopeptide quantification. The first set of experiments was conducted with wild-type Col-0 and the *kin10* mutant seedlings under NO donor (SNP) treatment or control conditions (Supplemental Table S3). Comparative analysis revealed that 3232 phosphosites showed significant changes (*p* < 0.05) between Col-0-SNP and Col-0-Control groups, including 2505 upregulated (>1.5-fold) and 727 downregulated (<0.67-fold) sites (Supplemental Fig. S1*B*). The *kin10*-SNP versus *kin10*-Control comparison identified 2788 significantly changed phosphosites, with 1546 upregulated and 1242 downregulated (Supplemental Fig. S6*A*). The significant reduction in SNP-induced phosphosites upregulation in the *kin10* mutant (2505 vs. 1546) suggests that *KIN10* partially mediates NO-dependent phosphorylation. Clustering analysis of phosphosites that were identified in both wild-type Col-0 and *kin10* mutant seedlings, and significantly upregulated by NO treatment in Col-0, revealed 4 distinct response patterns between Col-0 and *kin10* upon NO treatment (Fig. 5*C* and Supplemental Table S5). In clusters 1 and 3, phosphorylation levels increased upon NO treatment in both Col-0 and *kin10*. In cluster 2, phosphorylation levels were higher in *kin10* under control conditions compared to Col-0, while NO treatment induced an increase in Col-0 but not in *kin10*. In cluster 4, phosphorylation levels increased in response to NO in Col-0 but not in *kin10*, indicating *KIN10* dependence. GO and KEGG enrichment analyses of phosphoproteins in these clusters were consistent with known SnRK1/AMPK functions in energy homeostasis and RNA processing (56, 57, 58, 59, 60). Notably, spliceosome-related proteins and RNA-binding proteins were significantly enriched in clusters 2 and 4 (Fig. 5*C* and Supplemental Table S5), further implicating *KIN10* as a central regulator of NO signaling.

Given the functional redundancy between *KIN10* and *KIN11*, we next explored the role of *S*-nitrosylation at Cys-177 through quantitative phosphoproteomic analyses in a *kin10 kin11* double mutant background. To this end, we crossed *pKIN10*::*KIN10-GFP*/*kin10* or *pKIN10*::*KIN10*^*C177S*^*-GFP*/*kin10* transgenic plants with the *kin11* null mutant, generating *pKIN10*::*KIN10-GFP* or *pKIN10*::*KIN10*^*C177S*^*-GFP* transgenes in the *kin10 kin11* double mutant background, designated as *KIN10-GFP*/*kin10 kin11* and *KIN10*^*C177S*^*-GFP*/*kin10 kin11*, respectively. The second set of quantitative phosphoproteomic experiments was performed using *KIN10-GFP*/*kin10 kin11* and *KIN10*^*C177S*^*-GFP*/*kin10 kin11* under SNP treatment or control conditions (Supplemental Fig. S6, *B* and *C*, and Supplemental Table S6). Clustering analysis of phosphosites that were identified in both *KIN10-GFP*/*kin10 kin11* and *KIN10*^*C177S*^*-GFP*/*kin10 kin11* transgenic plants, and significantly upregulated in *KIN10-GFP*/*kin10 kin11* by NO treatment, revealed 4 distinct clusters between *KIN10-GFP*/*kin10 kin11* and *KIN10*^*C177S*^*-GFP*/*kin10 kin11* under NO treatment (Fig. 5*D* and Supplemental Table S7). In cluster 1, phosphorylation levels were higher in *KIN10*^*C177S*^*-GFP/kin10 kin11* under control conditions compared to *KIN10-GFP*/*kin10 kin11*, while NO treatment induced an increase in *KIN10-GFP*/*kin10 kin11* but not in *KIN10*^*C177S*^*-GFP*/*kin10 kin11*. In cluster 4, phosphorylation levels were comparable under control conditions but increased in response to NO in *KIN10-GFP*/*kin10 kin11* while remaining nearly unchanged in *KIN10*^*C177S*^*-GFP*/*kin10 kin11* (Fig. 5*D*). We further analyzed the enrichment of GO terms and KEGG pathways among the phosphoproteins in each cluster using Fisher’s exact test. Carbon fixation and carbohydrate metabolism pathways (KEGG) were significantly enriched in cluster 1, whereas mRNA-binding proteins and proteins involved in the regulation of defense response were enriched in cluster 4 (Fig. 5*D*). These findings suggest that *S*-nitrosylation at Cys-177 of KIN10 plays an important role in cellular energy homeostasis, gene expression regulation, and stress responses.

We then conducted an in-depth analysis of the role of Cys-177 in KIN10 in NO signaling by integrating two independent quantitative phosphoproteomic databases. Our results showed that a total of 8907 phosphosites were identified in both datasets, among which 1846 phosphosites were significantly induced by NO treatment in Col-0 (Col-0-SNP/Col-0-Control >1.5, *p* < 0.05). Among these, while 994 phosphosites were significantly more induced by NO treatment in Col-0 compared to *kin10* [(Col-0-SNP/Col-0-Control)/(*kin10*-SNP/*kin10*-Control) > 1.5], 343 phosphosites exhibited higher NO-induced phosphorylation in *KIN10-GFP*/*kin10 kin11* compared to *KIN10*^*C177S*^*-GFP*/*kin10 kin11* plants [(*KIN10-GFP*-SNP/*KIN10-GFP*-Control)/(*KIN10*^*C177S*^*-GFP*-SNP/*KIN10*^*C177S*^*-GFP*-Control) > 1.5] (Fig. 5*E* and Supplemental Table S8). Enrichment analysis of GO terms among the 161 phosphopeptides induced by NO treatment in both Col-0/*kin10* and *KIN10-GFP*/*KIN10*^*C177S*^*-GFP* groups indicated that *S*-nitrosylation of KIN10 at Cys-177 is involved in RNA splicing and the regulation of metabolic processes (Supplemental Fig. S6*D* and Supplemental Table S8), consistent with the established role of KIN10 in NO signaling (Fig. 5*C*). Together, these results indicate that Cys-177 plays a crucial role in KIN10’s ability to sense NO signals, thereby facilitating the transduction of signals to downstream targets.

### *S*-nitrosylation of KIN10 at Cys-177 is Involved in RNA Splicing

**Fig. 6:**
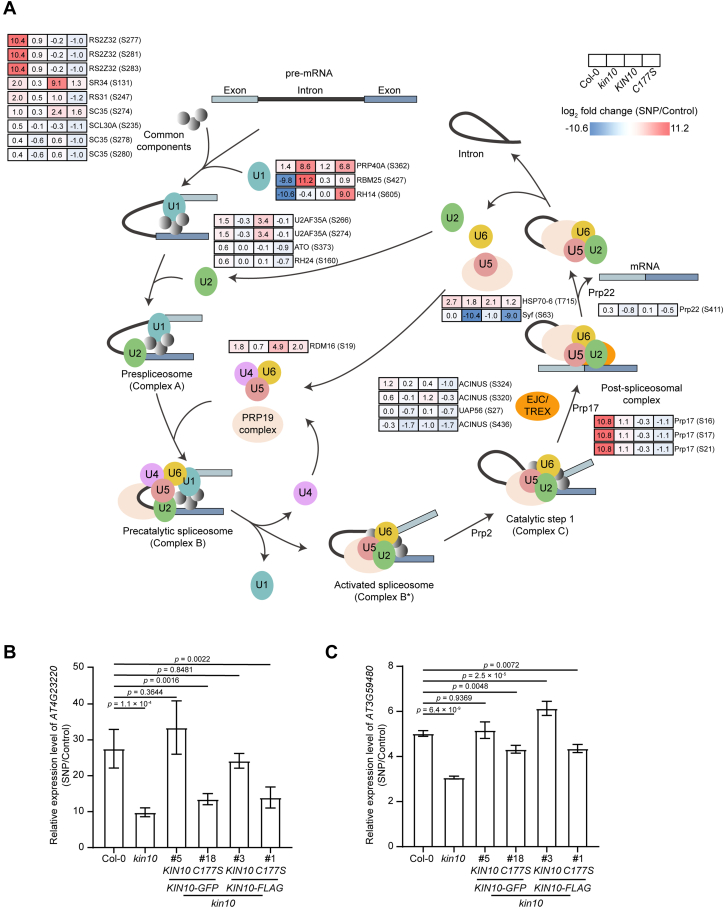
***S*-nitrosylation of KIN10 at Cys-177 regulates spliceosome component phosphorylation and gene expression.***A*, phosphorylation profiling of splicing factors within the spliceosome upon NO treatment. The diagram of the spliceosome pathway, modified from KEGG (ath03040), highlights phosphosites in splicing factors within the spliceosome that exhibit significant differential phosphorylation between WT (Col-0) and the *kin10* mutant, as well as between *KIN10-GFP* and *KIN10*^*C177S*^*-GFP* transgenic plants in the *kin10 kin11* double mutant background in response to NO. The log_2_ fold change (SNP/Control) for each phosphosite in the indicated genotypes is shown and color-coded accordingly. *B* and *C*, qRT-PCR analysis of *AT4G23220* (*B*) and *AT3G59480* (*C*) expression in wild-type (Col-0), *kin10* mutant, and *kin10* seedlings carrying *pKIN10*::*KIN10-GFP*, *pKIN10*::*KIN10^C177S^-GFP*, *pKIN10*::*KIN10-FLAG*, or *pKIN10*:*:KIN10^C177S^-FLAG* transgenes. Seedlings were germinated and grown on 1/2 MS agar plates under long-day conditions (16 h light/8 h dark). Six-day-old seedlings were treated with or without (control) 2 mM SNP applied on the lid of the Petri dishes for 3 h. The relative expression of *AT4G23220* or *AT3G59480* in Col-0 seedlings following SNP treatment, normalized to control conditions, is set as 1.0. Data are presented as mean ± SD, one-way ANOVA with Tukey’s HSD test.

The spliceosome is a dynamic RNA-protein complex responsible for the removal of introns from pre-mRNA transcripts, a process known as pre-mRNA splicing, which is essential for the regulation of eukaryotic gene expression. This macromolecular complex consists of five small nuclear ribonucleoproteins (snRNPs)–U1, U2, U4, U5, and U6–as well as numerous non-snRNP protein splicing factors. The phosphorylation of these splicing factors modulates protein-protein and protein-RNA interactions during spliceosome assembly and catalysis (74, 75, 76, 77). Given that *S*-nitrosylation of KIN10 is involved in the RNA splicing pathway, we subsequently focused on the phosphorylation of splicing factors in response to NO treatment. We found that a large number of splicing factors are regulated by NO through phosphorylation. In total, 344 phosphosites across splicing-related proteins were identified in both quantitative phosphoproteomic datasets, among which 108 sites exhibited significant phosphorylation changes in response to NO treatment between Col-0 and *kin10*. Further analysis revealed 27 phosphosites dependent on both *KIN10* and Cys-177 for NO responsiveness (Fig. 6*A* and Supplemental Table S9). These include five serine/arginine-rich (SR) proteins (RS2Z32, RS31, SCL30A, SC35, and SR34), as well as RNA helicases such as Prp17 and Prp22, proteins in the PRP19 complex, and other spliceosome components (Fig. 6*A*). These results suggest that NO regulates the phosphorylation of key spliceosome components via *S*-nitrosylation of KIN10 at Cys-177, thereby modulating RNA splicing processes.

Reversible phosphorylation of splicing factors within the spliceosome significantly contributes to pre-mRNA processing and the regulation of gene expression in eukaryotic cells (78). We next investigated genes that are regulated by both NO and *KIN10*. We found that *AT4G23220*, which encodes cysteine-rich receptor-like protein kinase 14, was significantly upregulated upon SNP treatment (Fig. 6*B*). However, this NO-induced upregulation was compromised in the *kin10* mutant. Notably, the reduced NO sensitivity of the *kin10* mutant was nearly fully restored by *KIN10-GFP* or *KIN10-FLAG* transgenes, but not by *KIN10*^*C177S*^*-GFP* or *KIN10*^*C177S*^*-FLAG* transgenes. Similarly, *AT3G59480*, a gene that encodes fructokinase-4, exhibited a similar NO- and KIN10-regulated pattern to that of *AT4G23220* (Fig. 6*C*). Collectively, these findings suggest that *S*-nitrosylation at Cys-177 of KIN10 is critical for NO-mediated gene expression regulation.

### Discussion

In this study, we present compelling evidence from genetic, biochemical, transcriptomic, and phosphoproteomic analyses demonstrating that NO-mediated *S*-nitrosylation at Cys-177 of KIN10, a catalytic α-subunit of SnRK1, inhibits its proteasomal degradation. This stabilization of KIN10 protein facilitates the transmission of NO signals to downstream targets, particularly the spliceosome, thereby regulating gene expression. As the mechanisms underlying NO-modulated gene expression remain largely unclear, our findings have important implications for further elucidating NO’s role in plant development and stress responses.

AMPK/SNF1/SnRK1 kinases are evolutionarily conserved energy sensors that are ubiquitous across eukaryotic organisms, ranging from simple unicellular fungi to complex animals and plants (22, 23). When confronted with stress and environmental fluctuations, plants typically respond by modulating redox homeostasis, a process mediated in part through the regulation of intracellular reactive nitrogen species, particularly NO (79). Nevertheless, the connection between NO signaling and energy homeostasis remains poorly understood. Data presented in this study reveal that NO signaling can modulate energy-sensing mechanisms through the *S*-nitrosylation of KIN10, potentially enabling plants to integrate redox status with energy homeostasis. Given that Cys-177 is absolutely conserved across the AMPK/SNF1/SnRK1 family, this mechanism of NO-mediated KIN10 *S*-nitrosylation may also be functional in other eukaryotic organisms.

Post-translational modification is a fundamental mechanism for regulating protein activity and stability. In addition to phosphorylation by upstream kinases, KIN10 undergoes other modifications, such as SUMOylation and ubiquitination (31, 33, 39). Notably, KIN10 activity is redox-sensitive and partially depends on a conserved cysteine residue–Cys-177–in its T-loop *in vitro* (42). The corresponding conserved residue in rat AMPKα2, Cys-174, undergoes oxidation in response to H_2_O_2_ treatment and plays a critical role in regulating AMPK activation (80). Collectively, these observations, together with our findings, highlight the importance of redox regulation in modulating the activity of KIN10, a central energy sensor, in response to internal and external cues. Furthermore, the divergent effects of H_2_O_2_-mediated oxidation versus NO-mediated *S*-nitrosylation at Cys-174/Cys-177 on AMPKα/KIN10 activity likely reflect the sophisticated fine-tuning of AMPK/SNF1/SnRK1 kinase regulation.

We observe that Cys-177 of KIN10 is positioned in close proximity to Thr-175, a critical residue whose phosphorylation serves as an essential prerequisite for SnRK1 kinase activity (26). This spatial relationship could lead to a hypothesis that the KIN10^C177S^ mutation might induce conformational changes that subsequently affect Thr-175 phosphorylation. Alternatively, the absence of *S*-nitrosylation at Cys-177 in KIN10^C177S^ might enhance its interaction with other components, such as the 26S proteasome, thereby accelerating its turnover and reducing KIN10 accumulation. Two lines of evidence support the latter hypothesis. First, KIN10 degradation is strictly dependent on its kinase activity, as two inactive variants–KIN10^K48M^ and KIN10^T175A^–accumulate to higher levels than wild-type KIN10 (26, 32, 33). In the cell-free degradation assay, both KIN10-FLAG and KIN10^C177S^-FLAG proteins were gradually degraded in the presence of CHX, whereas the KIN10^T175A^-FLAG protein remained stable. This observation suggests that Thr-175 phosphorylation still occurs in KIN10^C177S^ but not in KIN10^T175A^, thereby disfavoring the possibility that the KIN10^C177S^ mutation inhibits Thr-175 phosphorylation. Second, the KIN10^C177S^-GFP transgene rescued the lethal phenotype of the *kin10 kin11* double mutant, indicating that KIN10^C177S^ retains fundamental kinase function, unlike the inactive KIN10^T175A^. Collectively, these results suggest that the KIN10^C177S^ mutation does not directly impair Thr-175 phosphorylation and that the molecular mechanisms underlying the phenotypes of KIN10^C177S^ and KIN10^T175A^ are distinct.

Previous studies have established that NO exerts its physiological effects through post-translational modification and by influencing gene expression (4, 17). Consistent with these findings, we observed that NO treatment induced differential expression of numerous genes enriched in stress response and metabolic pathways. Notably, both the *kin10* mutant and *KIN10*^*C177S*^ transgenic plants exhibited abolished responsiveness to NO, indicating that KIN10 functions as an NO sensor, transducing signals via *S*-nitrosylation at Cys-177. RNA splicing factors, including SRRM1L, SRSF1, and GRP8, have been identified as direct targets of AMPK/SnRK1 (56, 57, 60). Our quantitative phosphoproteomic analyses demonstrated that *S*-nitrosylation of KIN10 at Cys-177 contributes to the phosphorylation of RNA splicing factors within the spliceosome, potentially representing a significant mechanism by which NO modulates gene expression. Since abiotic stress enhanced the *S*-nitrosylation of KIN10, we speculate that this post-translational modification of KIN10 is involved in regulating the expression of stress-responsive genes. Two genes, *AT4G23220* and *AT3G59480*, which are involved in plant growth and carbohydrate metabolism, respectively, are regulated by both NO and *S*-nitrosylation of KIN10, further supporting the notion that KIN10 serves as a critical regulator of NO signaling in energy homeostasis and gene expression. Collectively, our results reveal that *S*-nitrosylation of KIN10 at Cys-177 is crucial for transmitting NO signals to downstream targets, which may regulate gene expression through modulation of spliceosomal component phosphorylation. Elucidating the specific splicing events regulated by NO-KIN10 signaling remains a major challenge for future research.

### Data Availability

The raw RNA-seq and mass spectrometry proteomics data generated in this study, along with the search result files, have been deposited at the National Genomics Data Center, China National Center for Bioinformation/Beijing Institute of Genomics, Chinese Academy of Sciences (81). RNA-seq data are available in the Genome Sequence Archive under accession number CRA025666 (https://ngdc.cncb.ac.cn/gsa/browse/CRA025666). Proteomics data are available in OMIX under accession numbers OMIX010149 (https://ngdc.cncb.ac.cn/omix/release/OMIX010149), OMIX010152 (https://ngdc.cncb.ac.cn/omix/release/OMIX010152), and OMIX010155 (https://ngdc.cncb.ac.cn/omix/release/OMIX010155).

#### Supporting information

This article contains supporting information.

## Conflict of interest

The authors declare no competing interests.
