# Supplementary material for: Nitric Oxide-mediated S-nitrosylation of the Energy Sensor KIN10 Regulates RNA Splicing and Gene Expression in Arabidopsis
Source: Mol Cell Proteomics. 2025 Nov 25;25(1):101459. doi: 10.1016/j.mcpro.2025.101459 (PMC12799962; doi:10.1016/j.mcpro.2025.101459)
Supplement: Table S10 [file mmc10.docx]

Table S10 Primers used in this study

| Primer | Sequence (5'-3') | Experiment |
| --- | --- | --- |
| ACT7 qRT-F | TCCATGAAACAACTTACAACTCCATCA | qRT-PCR |
| ACT7 qRT-R | CATCGTACTCACTCTTTGAAATCCACA | qRT-PCR |
| KIN10 qRT-F | GGAGATGGAGGAGAAAGTGAG | qRT-PCR |
| KIN10 qRT-R | GAGTTCACATACTCCATGACAAG | qRT-PCR |
| *kin10*-LP | cagttacaaaaatggccatgg | *kin10* genotyping |
| *kin10*-RP | tgaaacgagtaaccatccctg | *kin10* genotyping |
| LBb1.3 | ATTTTGCCGATTTCGGAAC | *kin10* genotyping |
| WiscDsLox384F5-LP | CGTAGTGATCCACATGTGCAG | *kin11* genotyping |
| WiscDsLox384F5-RP | GATTGCAGACTTTGGGTTGAG | *kin11* genotyping |
| 8474 | ATAATAACGCTGCGGACATCTACATTTT | *kin11* genotyping |
| proKIN10::KIN10-F | TGAGGTTTCCGGAATTCCCTT | pGEM-T-proKIN10::KIN10 |
| proKIN10::KIN10-R | TTCAGAGGACTCGGAGCTGA | pGEM-T-proKIN10::KIN10 |
| proKIN10::KIN10-FLAG-F | AAGCTTACTAGTGGATCCATCGATAGTACTGTCGACTGAGGTTTCCGGAATTCCCTT | pCAMBIA1300-proKIN10::KIN10-FLAG |
| proKIN10::KIN10-FLAG-R | TAATCACCGTCATGGTCTTTGTAGTCCATCCCGGGGAGGACTCGGAGCTGAGCAAGAAA | pCAMBIA1300-proKIN10::KIN10-FLAG |
| proKIN10::KIN10-GFP-F | AAACGACGGCCAGTGCCAAGCTTCTCGAGGTCGACTGAGGTTTCCGGAATTCCCTT | pCAMBIA1300-proKIN10::KIN10-GFP |
| proKIN10::KIN10-GFP-R | CCAGTGAAAAGTTCTTCTCCTTTACCAGCCCCGGGGAGGACTCGGAGCTGAGCAAGAAA | pCAMBIA1300-proKIN10::KIN10-GFP |
| C133S-F | CAGGAGTGGAATACTCCCATCGAAAC | KIN10^C133S^ mutant |
| C133S-R | GAGTATTCCACTCCTGATATTATCT | KIN10^C133S^ mutant |
| C177S-F | TTTTGAAGACAAGTTCTGGAAGTCCA | KIN10^C177S^ mutant |
| C177S-R | GAACTTGTCTTCAAAAAATGACCAT | KIN10^C177S^ mutant |
| T175A-F | GGTCATTTTTTGAAGGCAAGTTGTGG | KIN10^T175A^ mutant |
| T175A-R | CCTTCAAAAAATGACCATCTCGCATT | KIN10^T175A^ mutant |
