# Supplementary material for: Nitric Oxide-mediated S-nitrosylation of the Energy Sensor KIN10 Regulates RNA Splicing and Gene Expression in Arabidopsis
Source: Mol Cell Proteomics. 2025 Nov 25;25(1):101459. doi: 10.1016/j.mcpro.2025.101459 (PMC12799962; doi:10.1016/j.mcpro.2025.101459)
Supplement: Supplemental data [file mmc11.pdf]

Nitric oxide-mediated *S*-nitrosylation of the energy sensor KIN10 regulates RNA splicing and gene expression in *Arabidopsis*

Yanyan Yi,[1,2,‡] Xiahe Huang,[3,‡] Wan Wang,[1] Yingchun Wang,[2,3] Jianru Zuo,[1,2] Hongyan Guo[1,*]

List of the material included:

Figure S1. NO regulates metabolism and RNA splicing in Arabidopsis

Figure S2. Sequence alignment of KIN10-like proteins from different species

Figure S3. Cys-133 and Cys-418 of KIN10 are *S*-nitrosylated

Figure S4. NO positively regulates KIN10 stability

Figure S5. *S*-nitrosylation of KIN10 at Cys-177 positively regulates protein stability

Figure S6. *S*-nitrosylation at Cys-177 of KIN10 is involved in RNA splicing

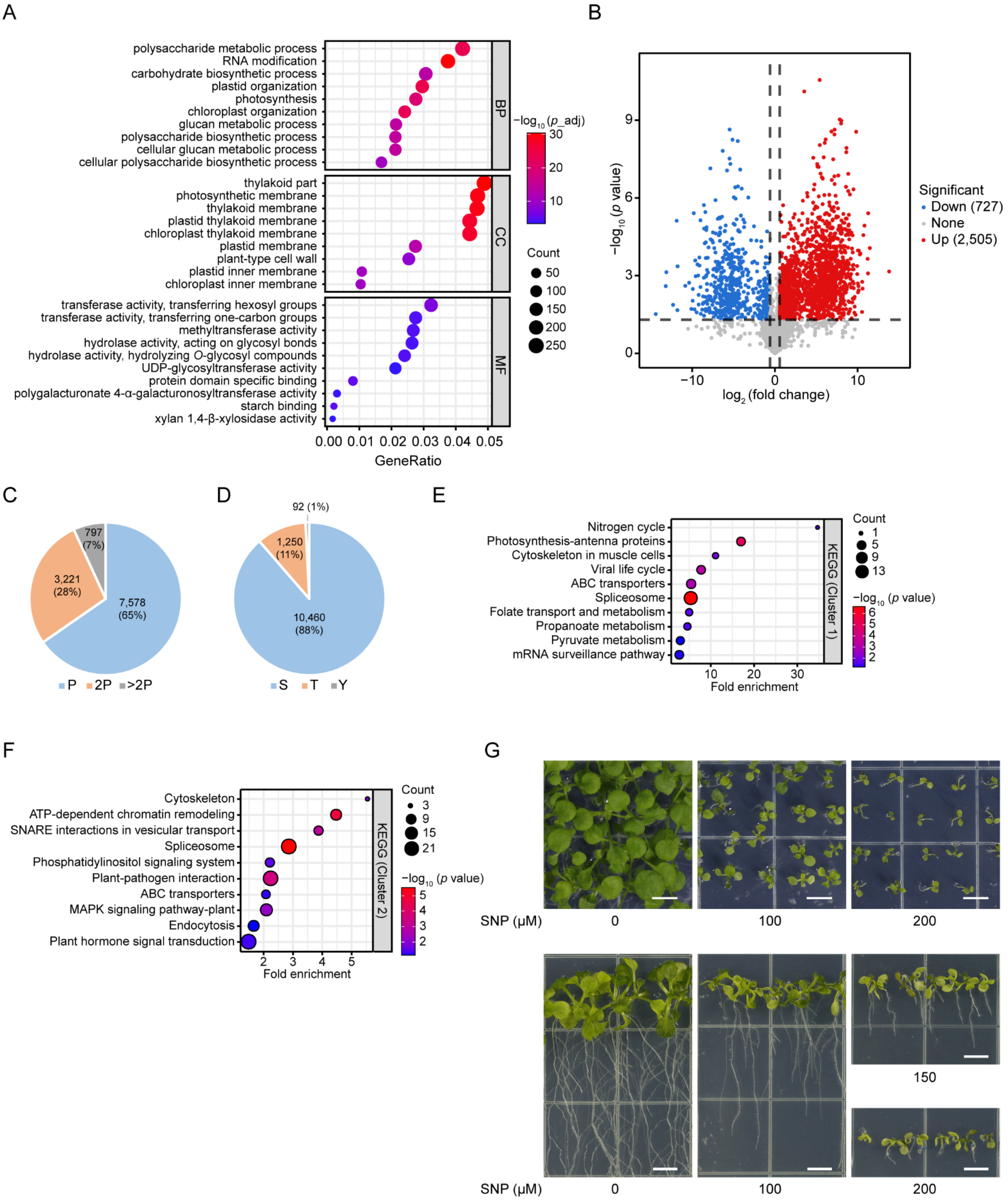

Figure S1. NO regulates metabolism and RNA splicing in Arabidopsis

(A) Top significantly enriched Gene Ontology terms for biological process (BP), cellular component (CC), and molecular function (MF) categories derived from the downregulated DEGs shown in Figure 1A.

(B) Volcano plot showing changes in phosphosites between sodium nitroprusside (SNP) treatment and control conditions. Twelve-day-old wild-type (Col-0) seedlings were treated with or without

(control) 2 mM SNP applied on the lid of the Petri dishes for 5 h. Red (upregulated) and blue (downregulated) dots represent significantly changed phosphosites, with thresholds of > 1.5-fold or < 0.67-fold change in SNP treatment versus control conditions ($p < 0.05$).

(C) Percentages and numbers of singly (P), doubly (2P), and multiply (>2P) phosphorylated peptides identified in the assay.

(D) Distribution of phosphorylated amino acid residues (serine–S, threonine–T, tyrosine–Y) identified in the assay.

(E) Top significantly enriched KEGG pathways derived from phosphosites in cluster 1 shown in Figure 1C ($p < 0.05$).

(F) Top significantly enriched KEGG pathways derived from phosphosites in cluster 2 shown in Figure 1C ($p < 0.05$).

(G) Two-week-old wild-type (Col-0) seedlings germinated and grown horizontally (top) or vertically (bottom) on 1/2 MS agar plates with or without SNP as indicated. Scale bars, 0.5 cm.

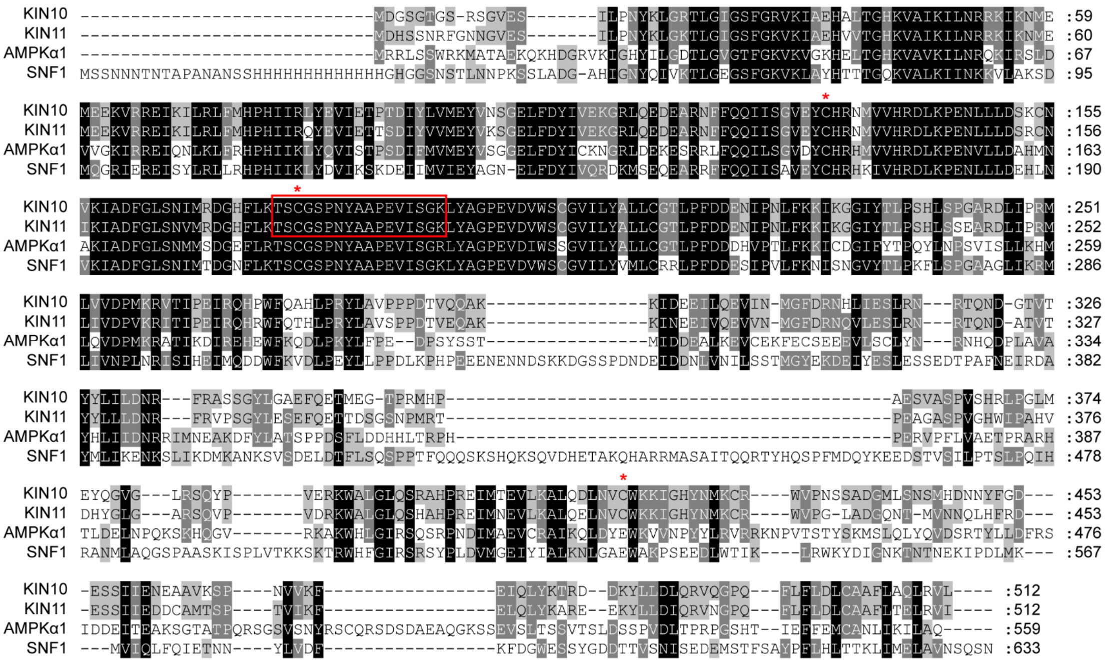

Figure S2. Sequence alignment of KIN10-like proteins from different species

Cysteine residues identified as *S*-nitrosylated in GSNO-treated MBP-KIN10 recombinant protein by liquid chromatography-tandem mass spectrometry (LC-MS/MS) are marked with red asterisks. The peptide identified in a site-specific nitrosoproteomics study is highlighted with a red rectangle. The protein sequences used for alignment are as follows, with their respective accession numbers: *Arabidopsis thaliana* KIN10: NP_566130.1; *Arabidopsis thaliana* KIN11: NP_566843.1; *Homo sapiens* AMPKα1: NP_006242.5; *Saccharomyces cerevisiae* SNF1: NP_010765.3. Sequence alignment was performed using Clustal X2 and Genedoc.

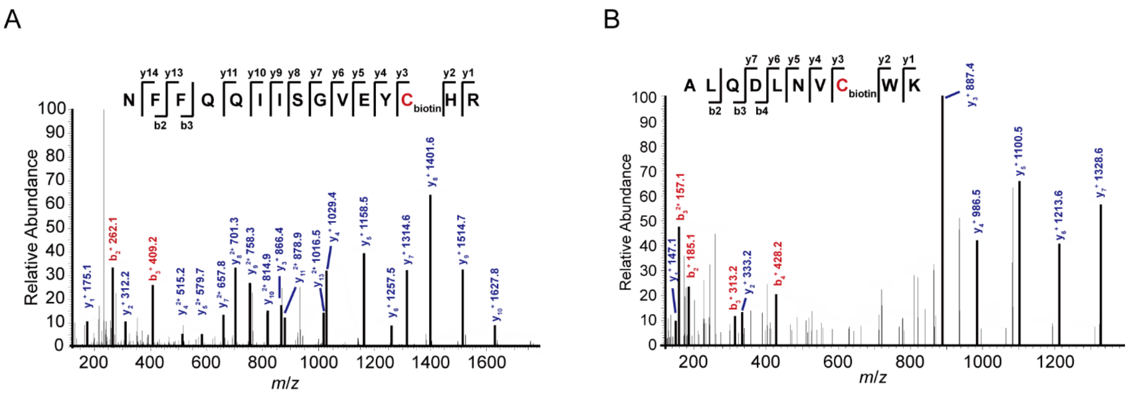

Figure S3. Cys-133 and Cys-418 of KIN10 are *S*-nitrosylated

Mass spectrometric analysis of tryptic fragments from GSNO-treated MBP-KIN10 recombinant protein. The b- and y-type product ions are indicated, with Cys-133 (A) and Cys-418 (B) identified as *S*-nitrosylated residues labeled with biotin.

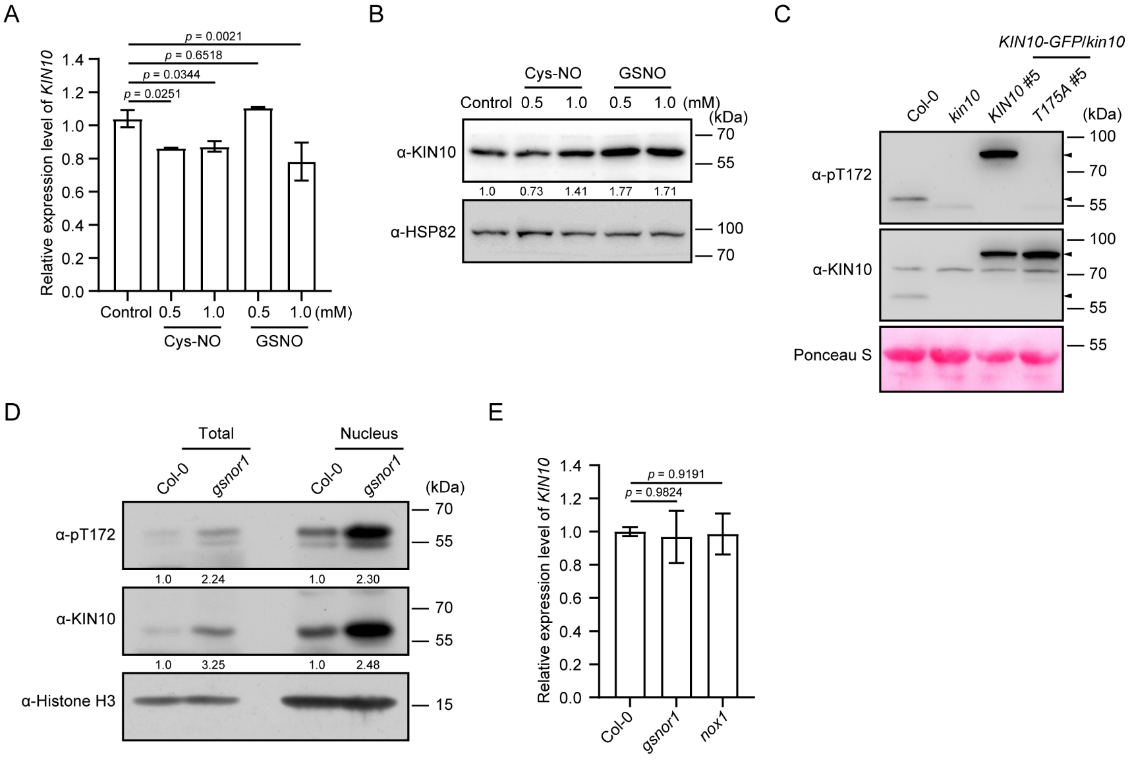

Figure S4. NO positively regulates KIN10 stability

(A) Quantitative RT-PCR (qRT-PCR) analysis of *KIN10* expression in 10-day-old wild-type (Col-0) seedlings treated with or without (control) Cys-NO or GSNO for 10 h. The relative expression of *KIN10* in control Col-0 seedlings is set as 1.0. Data are presented as mean ± SD, one-way ANOVA with Tukey's HSD test.

(B) Immunoblotting analysis of KIN10 protein levels in 10-day-old Col-0 seedlings treated with or without (control) Cys-NO or GSNO for 10 h. Quantification is shown below the blot. The relative level of KIN10 protein normalized to HSP82 in the control sample is set as 1.0.

(C) Analysis of the specificity of the KIN10 and pT172 antibodies. The *kin10* mutant and *kin10* seedlings carrying *pKIN10*::$KIN10^{T175A}$*-GFP* transgene served as negative controls.

(D) Analysis of KIN10 phosphorylation and accumulation in total and nuclear fractions of 2-week-old Col-0 and *gsnor1* mutant seedlings using anti-pT172 and anti-KIN10 antibodies, respectively. Histone H3 was used as a nuclear marker. The relative levels of phosphorylated KIN10 and total KIN10 protein normalized to Histone H3 in Col-0 seedlings are set as 1.0, respectively.

(E) Analysis of *KIN10* expression in 12-day-old Col-0, *gsnor1*, and *nox1* mutant seedlings by qRT-PCR. The relative expression of *KIN10* in Col-0 seedlings is set as 1.0. Data are presented as mean ± SD, one-way ANOVA with Tukey's HSD test.

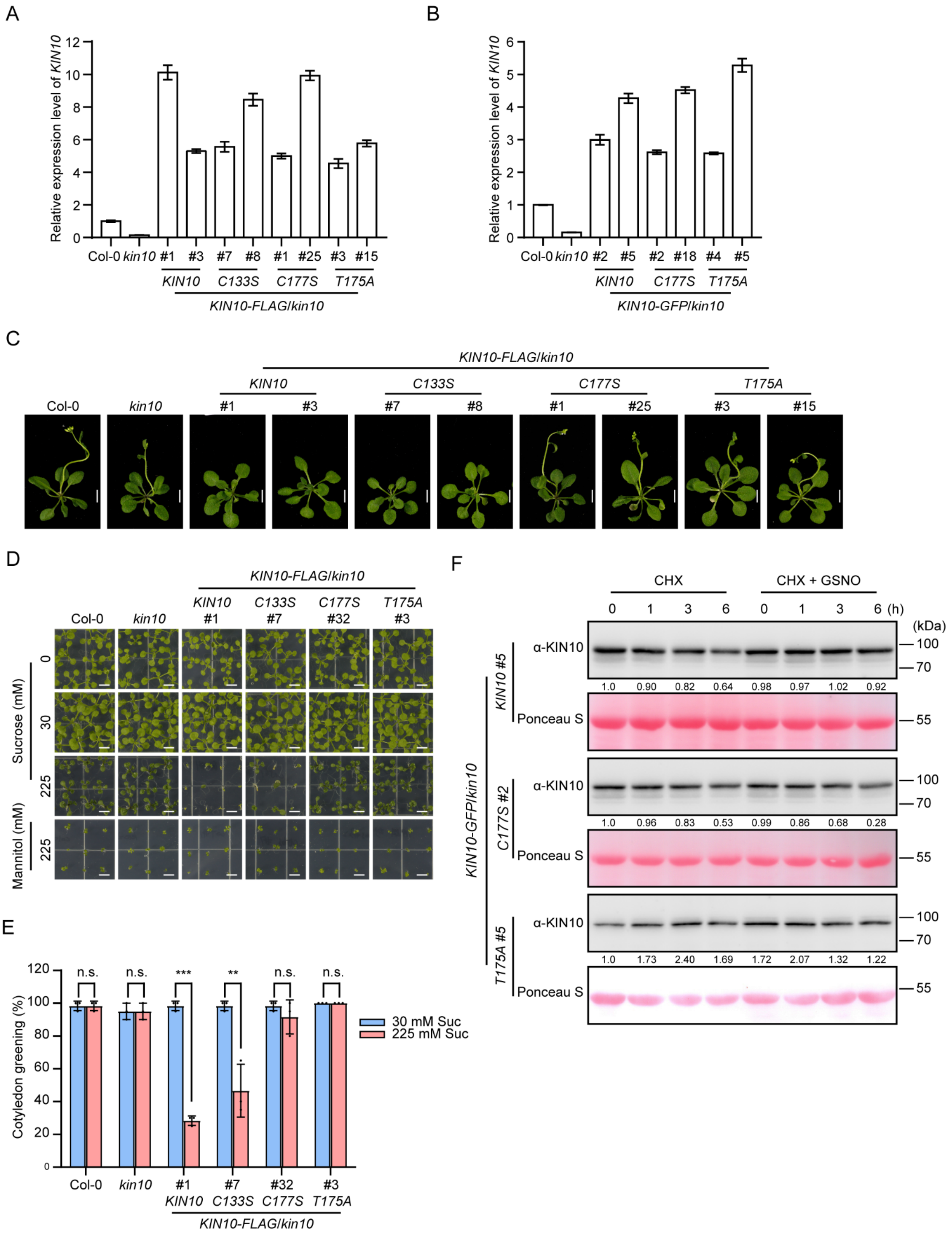

Figure S5. *S*-nitrosylation of KIN10 at Cys-177 positively regulates protein stability

(A) Analysis of *KIN10* expression in 8-day-old wild-type (Col-0), the *kin10* mutant, and *kin10* seedlings carrying *pKIN10*::*KIN10-FLAG*, *pKIN10*::*KIN10*^{C133S}*-FLAG*, *pKIN10*::*KIN10*^{C177S}-

FLAG, or *pKIN10*::*KIN10*^{T175A}-*FLAG* transgenes by quantitative RT-PCR (qRT-PCR). Transgenic line numbers are indicated. The relative expression of *KIN10* in Col-0 seedlings is set as 1.0.

(B) Analysis of *KIN10* expression in 8-day-old Col-0, the *kin10* mutant, and *kin10* seedlings carrying *pKIN10*::*KIN10-GFP*, *pKIN10*::*KIN10*^{C177S}-*GFP*, or *pKIN10*::*KIN10*^{T175A}-*GFP* transgenes by qRT-PCR. Transgenic line numbers are indicated. The relative expression of *KIN10* in Col-0 seedlings is set as 1.0.

(C) Three-week-old seedlings with the indicated genotypes germinated and grown on 1/2 MS agar plates under long-day conditions (16 h light/8 h dark). Scale bars, 0.5 cm.

(D) Two-week-old seedlings with the indicated genotypes germinated and grown on 1/2 MS agar plates supplemented with or without sucrose or mannitol as indicated. Scale bars, 0.5 cm.

(E) Quantitative analysis of the cotyledon greening rate for seedlings shown in (D) (n = 60). Data are presented as mean ± SD, two-tailed Student's t-test, *** $p < 0.001$, ** $p < 0.01$, n.s. indicates not significant.

(F) Cell-free protein degradation assay of KIN10 protein in extracts from *pKIN10*::*KIN10-GFP*, *pKIN10*::*KIN10*^{C177S}-*GFP*, and *pKIN10*::*KIN10*^{T175A}-*GFP* transgenic seedlings in the *kin10* mutant background. Protein extracts from 10-day-old transgenic seedlings were incubated with 100 μM CHX in the presence or absence of 100 μM GSNO. Samples were collected at the indicated time points. Ponceau S staining was used as a loading control. Quantification is shown below the blot. The relative level of KIN10-GFP normalized to the Ponceau S-stained Rubisco large subunit in the untreated sample is set as 1.0.

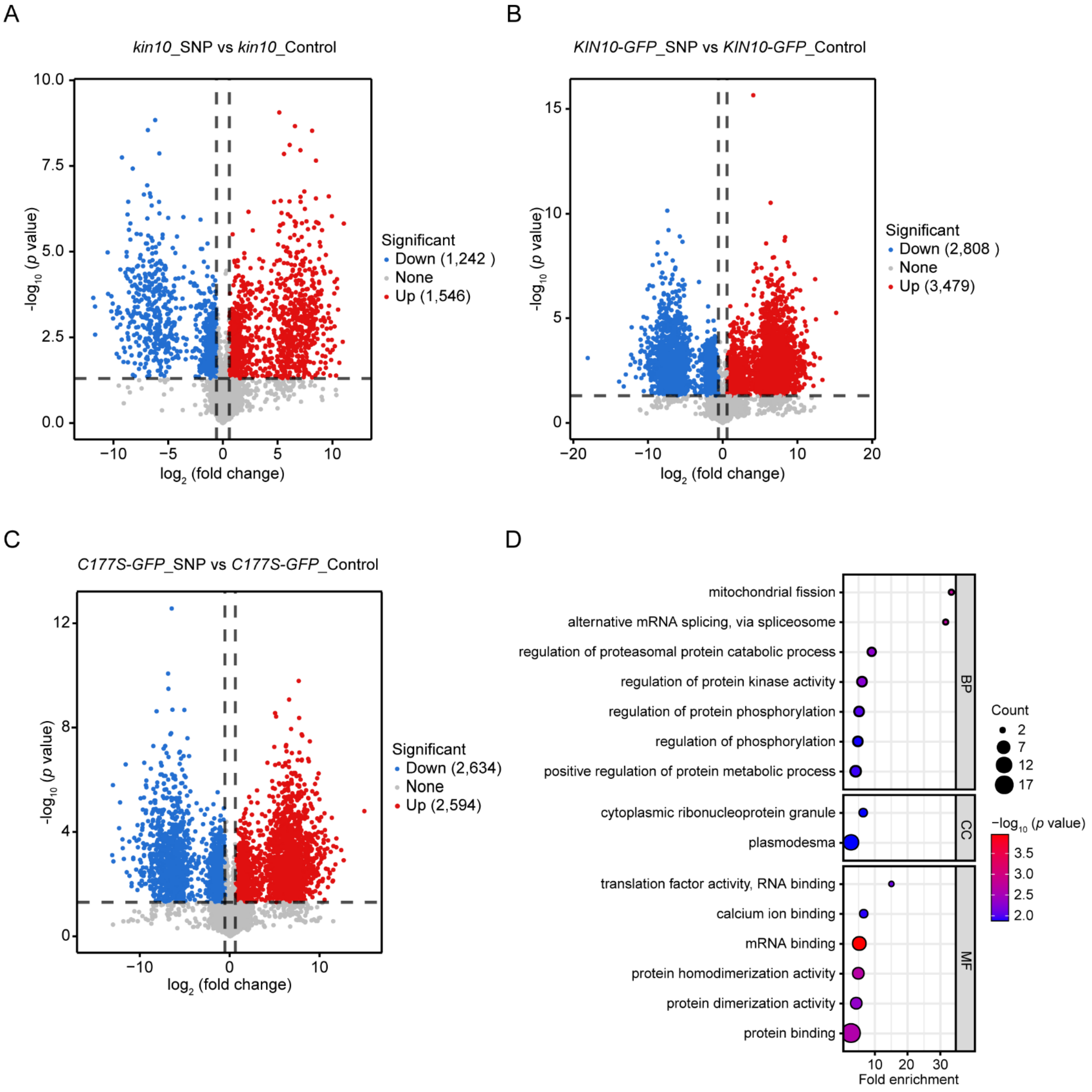

Figure S6. *S*-nitrosylation at Cys-177 of KIN10 is involved in RNA splicing

(A–C) Volcano plots showing changes in phosphosites between sodium nitroprusside (SNP) treatment and control conditions. Twelve-day-old *kin10* mutant (A), *kin10 kin11* seedlings carrying *pKIN10*::*KIN10-GFP* (B) or *pKIN10*::*KIN10*^{C177S}*-GFP* (C) transgenes were treated with or without (control) 2 mM SNP applied on the lid of the Petri dishes for 5 h. Red (upregulated) and blue (downregulated) dots represent significantly changed phosphosites, with thresholds of > 1.5-fold or < 0.67-fold change in SNP treatment versus control conditions ($p < 0.05$).

(D) Significantly enriched Gene Ontology terms for biological process (BP), cellular component (CC), and molecular function (MF) categories, derived from 161 upregulated phosphopeptides that are dependent on *KIN10* and *S*-nitrosylation at Cys-177 upon NO treatment, as shown in Figure 5E.
